# Supplementary material for: Quantifying unmet need in General Practice: a retrospective cohort study of administrative data
Source: BMJ Open. 2023 Sep 15;13(9):e068720. doi: 10.1136/bmjopen-2022-068720 (PMC10510933; doi:10.1136/bmjopen-2022-068720)
Supplement: Author's manuscript [file bmjopen-2022-068720.draft_revisions.pdf]

BMJ Open

BMJ Open is committed to open peer review. As part of this commitment we make the peer review history of every article we publish publicly available.

When an article is published we post the peer reviewers' comments and the authors' responses online. We also post the versions of the paper that were used during peer review. These are the versions that the peer review comments apply to.

The versions of the paper that follow are the versions that were submitted during the peer review process. They are not the versions of record or the final published versions. They should not be cited or distributed as the published version of this manuscript.

BMJ Open is an open access journal and the full, final, typeset and author-corrected version of record of the manuscript is available on our site with no access controls, subscription charges or pay-per-view fees (http://bmjopen.bmj.com).

If you have any questions on BMJ Open's open peer review process please email info.bmjopen@bmj.com

BMJ Open

Quantifying unmet need in General Practice: a retrospective cohort study of administrative data

Journal:	*BMJ Open*
Manuscript ID	bmjopen-2022-068720
Article Type:	Original research
Date Submitted by the Author:	27-Sep-2022
Complete List of Authors:	McConnachie, Alex; University of Glasgow<br>Ellis, David; University of Bath, School of Management<br>Wilson, Philip; University of Aberdeen, Institute of Health and Wellbeing<br>McQueenie, Ross; University of Glasgow, Genera Practice and Primary Care<br>Williamson, Andrea; University of Glasgow, GPPC, School of Medicine, Dentistry and Nursing, MVLS
Keywords:	PRIMARY CARE, Organisation of health services < HEALTH SERVICES ADMINISTRATION & MANAGEMENT, Quality in health care < HEALTH SERVICES ADMINISTRATION & MANAGEMENT

SCHOLARONE™
Manuscripts

I, the Submitting Author has the right to grant and does grant on behalf of all authors of the Work (as defined in the below author licence), an exclusive licence and/or a non-exclusive licence for contributions from authors who are: i) UK Crown employees; ii) where BMJ has agreed a CC-BY licence shall apply, and/or iii) in accordance with the terms applicable for US Federal Government officers or employees acting as part of their official duties; on a worldwide, perpetual, irrevocable, royalty-free basis to BMJ Publishing Group Ltd ("BMJ") its licensees and where the relevant Journal is co-owned by BMJ to the co-owners of the Journal, to publish the Work in this journal and any other BMJ products and to exploit all rights, as set out in our licence.

The Submitting Author accepts and understands that any supply made under these terms is made by BMJ to the Submitting Author unless you are acting as an employee on behalf of your employer or a postgraduate student of an affiliated institution which is paying any applicable article publishing charge ("APC") for Open Access articles. Where the Submitting Author wishes to make the Work available on an Open Access basis (and intends to pay the relevant APC), the terms of reuse of such Open Access shall be governed by a Creative Commons licence – details of these licences and which Creative Commons licence will apply to this Work are set out in our licence referred to above.

Other than as permitted in any relevant BMJ Author's Self Archiving Policies, I confirm this Work has not been accepted for publication elsewhere, is not being considered for publication elsewhere and does not duplicate material already published. I confirm all authors consent to publication of this Work and authorise the granting of this licence.

For peer review only

Quantifying unmet need in General Practice: a retrospective cohort study of administrative data

Alex McConnachie[1], David Ellis[2], Philip Wilson[3], Ross McQueenie[4], Andrea E Williamson[5]

[1]BSc MSc PhD, Professor of Clinical Trial Biostatistics, Robertson Centre for Biostatistics, School of Health and Wellbeing, MVLS, University of Glasgow, Level 11 Boyd Orr Building, University Avenue, Glasgow G12 8QQ, UK. ORCID ID: 0000-0002-7262-7000

[2] MA MSc PhD, Associate Professor in Information Systems; School of Management, University of Bath, Claverton Down, Bath BA2 7AY, UK. ORCID ID: 0000-0001-6172-3323

[3] MA MB BChir DPhil DCH FRCPCH FRCGP, Professor of Primary Care and Rural Health, Centre for Rural Health, Institute of Applied Health Sciences, University of Aberdeen, Old Perth Road, Inverness IV2 3JH, UK. ORCID ID: 0000-0002-4123-8248

[4] BSc Hons, PhD, Research Associate, General Practice and Primary Care, School of Health and Wellbeing, MVLS, University of Glasgow, 1 Horselethill Road, Glasgow, G12 9LX UK. ORCID ID: 0000-0002-0070-6607

[5] MBChB MPH PhD FRCGP FHEA DTM&H, Professor of General Practice and Inclusion Health, General Practice and Primary Care, School of Health and Wellbeing, MVLS, University of Glasgow, 1 Horselethill Road, Glasgow, G12 9LX UK. ORCID ID: 0000-0002-8981-9068 @aewilliamsonl

Correspondence to: Professor Alex McConnachie, Robertson Centre for Biostatistics, Institute of Health and Wellbeing, MVLS, University of Glasgow, Level 11 Boyd Orr Building, University Avenue, UK, alex.mcconnachie@glasgow.ac.uk

Word count (excluding abstract, references, tables, figures): 2910

For peer review only

Abstract

Objectives: To assess whether patients attending General Practices (GPs) in socioeconomically deprived areas receive the same level of care, compared to similar patients (based on age, sex, and level of morbidity) attending GPs in less deprived areas. If not, to quantify the additional resource that would be required by GPs in deprived areas to achieve parity.

Design: Retrospective cohort study.

Setting: 150 General Practices in Scotland, UK, divided into two groups: 80 practices in Scottish Index of Multiple Deprivation (SIMD) decile 1-5 (more SE deprived); 70 practices in SIMD decile 6-10 (less SE deprived).

Patients: 437,590 patients registered with a more SE deprived GP, and 333,994 patients registered with a less SE deprived GP, for the whole study period (2013-2016), who made at least one appointment.

Outcomes: The number of contacts and total contact time between patients and clinical staff.

Results: Patients in more SE deprived areas had slightly more discrete contacts over 3 years (11.8 vs. 11.4), but each patient had marginally less contact time (146.1 vs 149.5 minutes). Stratified by sex and age, differences were also small. Stratified by the number of Long-Term Conditions (nLTCs), practices in more SE deprived areas delivered significantly less contact time than practices in less SE deprived areas. Over three years, 8 fewer minutes for patients with no LTCs, and 24, 27, 38, and 28 fewer minutes for patients with 1, 2, 3-4, or 5+ LTCs, respectively.

Conclusion: If GPs in more SE deprived areas were to give an equal amount of direct contact time to patients with the same level of need served by GPs in less SE deprived areas, this would require a 14% increase in patient contact time. This represents a significant unmet need, supporting the case for redistribution of resources to tackle the inverse care law.

Keywords: general practice, health care planning, unmet need, inverse care law, inequalities, big data

For peer review only - http://bmjopen.bmj.com/site/about/guidelines.xhtml

Strengths and limitations

Strengths

- Large dataset, based on routine administrative records, demonstrating the feasibility of measuring contact time with practice staff.
- Large sample of practices covering urban and rural, affluent and deprived areas.

Limitations

- The accuracy of estimated time spent with patients has not been proved.
- Health care need is estimated by a simple count of the number of long-term conditions, which does not reflect variation in health care needs between conditions, nor variation in needs between patients within the same condition.
- These data predate the rapid and far reaching changes to general practice access in the UK triggered by the Covid pandemic.

Study Protocol

See protocol paper.[11]

Funding

This work was supported by a grant from Chief Scientist Office, Scottish Government (reference CZH/4/1118) with Safe Haven and data linkage costs supported in lieu by the DSLS at Scottish Government. The funding sources for this analysis had no influence over study design, data collection, data analysis, data interpretation, the writing of the report, or the decision to submit for publication.

Competing Interests

None declared

For peer review only

For peer review only - http://bmjopen.bmj.com/site/about/guidelines.xhtml

Introduction

Unmet need is a multidimensional, complex construct;[1] "*when an individual does not receive an available and effective treatment that could have improved her health…some unmet need is acceptable since resources are scarce. What is of concern here is whether unmet need is inequitable, or systematically related to socioeconomic or other personal characteristics*".[2]

Patients living with multi-morbidity (two or more co-existing long-term health conditions (LTCs)) have complex needs, and successfully meeting those within our healthcare systems is one of the most pressing challenges of our time.[3-5] We already have strong evidence that people living in more socio-economically (SE) deprived areas in the UK have a burden of multi-morbidity at an earlier age.[6] Furthermore, patients with multi-morbidity are at greater risk of all-cause mortality[7, 8] and hospitalisation,[8] suggesting that the numbers of LTCs recorded in general practice electronic health records broadly reflect underlying healthcare need.

The analyses presented in this paper stem from research exploring the determinants and outcomes of missed appointments in general practice in Scotland. During this work, we determined the total number and duration of contacts between clinical practice staff and individual patients. We postulated that patients with more LTCs have greater healthcare need, and therefore require more contact time with clinical practice staff. In an equitable health service, equivalent patients (according to their age, sex, and number of LTCs) should, on average, receive the same level of contact with clinical practice staff, regardless of whether they live in an affluent or socio-economically (SE) deprived area. Therefore, we hypothesised that patients in SE deprived communities may receive less clinical GP staff contact time with an equivalent level of morbidity. If this is the case, how much more contact time would be required in socio-economically deprived practices to achieve parity with affluent practices?

For peer review only

For peer review only - http://bmjopen.bmj.com/site/about/guidelines.xhtml

Methods

A Trusted Third party (TTP) for the National Health Service (NHS) in Scotland (Albasoft), recruited general practices (GPs) on our behalf. A TTP is an organisation independent from the NHS with the skills, infrastructure and permissions to extract and link data safely without having a direct interest in the use to which the data will be put. The TTP is required to ensure confidentiality for patients and professionals in the research process.[9] The TTP recruited practices to ensure a spread of practices in urban and rural locations, serving both affluent and socio-economically (SE) deprived (Deep End) populations. All patients who were alive and registered at the same practice for the 3-year period (September 2013 to September 2016) and who made at least one appointment with the practice were included in the data set. All patients retained also had data on age, sex, number of recorded LTCs, and the number and total duration of all in person contacts with clinical practice staff such as GPs and practice nurses. Recording of patient ethnicity and language interpreter requirements was very low so this could not be included in our analysis.[10]

How we defined and measured LTCs are documented in previous publications.[10-12]

We present the number and percentage of patients in practices in affluent and deprived areas, according to sex, age (0-39, 40-59, 60-79, and 80+ years), and the number of LTCs (none, 1, 2, 3-4, or 5+). Practices were categorised by Scottish Index of Multiple Deprivation (SIMD), which is based on the average SIMD score from all patients registered at the practice. SIMD is routinely used in Scotland as part of the current GP contract funding allocation formula. When split by decile groups, 1 is the most SE deprived.[13]

We calculated the total number and duration of contacts in minutes for each patient. These were extracted from appointments recorded via practice IT systems. The duration of contacts was calculated from the 'in' and 'out' time. Detailed proof of concept work in our pilot study of 67,705 patient records enabled us to set rules about included appointments to increase accuracy.

For peer review only - http://bmjopen.bmj.com/site/about/guidelines.xhtml

Appointments that were 'open' for less than 2 minutes, 'open' for more than 24 hours, labelled as an administrative slot, or were not face-to-face appointments were excluded.[11] There is no unified categorisation of General Practice appointment types in the UK and this was reflected in the heterogeneity of appointment categories in our data set. Therefore, all appointments included were for face-to-face appointments with a health care professional in the practice (rather than being specific e.g. about GP or practice nurse contacts).[10]

We present the mean and standard deviation (SD) of the number and duration of contacts for patients in practices in affluent and deprived areas, according to sex, age, and the number of LTCs.

Taking the mean contact time provided by practices in affluent areas and applying this to the number of patients served by practices in SE deprived areas, we calculated the change in total contact time that would be required in practices in SE deprived areas in order to give the same average contact time. We then repeated this analysis, stratified by sex, age, and/or number of LTCs.

Results

Data from 150 Scottish practices were included. These were split into two groups: SIMD decile 1-5 (most SE deprived, 80 practices) and SIMD decile 6-10 (least deprived, 70 practices). From a cohort of 824,374 patients, after exclusion of patients who moved practice, or who had missing data for age, sex, or number of LTCs, data from 771,584 patient records were analysed. The study population was 437,590 patients from the 80 practices in SE deprived areas, and 333,994 patients from the 70 practices in affluent areas. **Table 1** sets out their characteristics. Differences in terms of the sex and age distributions are small. 47% of patients from SE deprived practices in this sample were male, compared to 46% of patients in practices in more affluent areas. Patients registered with practices in SE deprived areas were slightly younger, with 48% vs. 47% being under 40, and 4.6% vs. 5.2% being over 80 years of age. The difference in terms of the distribution of the number of LTCs that patients

For peer review only - http://bmjopen.bmj.com/site/about/guidelines.xhtml

For peer review only

142 had was far more marked, with 45% of patients in practices serving affluent populations having no LTCs, compared to 37% of patients in practices serving SE deprived populations.

Table 1: Study population. Numbers and percentages of patients in groups defined by sex, age, and number of long-term conditions, for practices with SE deprived and affluent populations.

		Practices in Deprived Areas	Practices in Affluent Areas
Number of Practices		80	70
Number of Patients		437590	333994
Sex			
Male	N (%)	205914 (47.1%)	155289 (46.5%)
Female	N (%)	231676 (52.9%)	178705 (53.5%)
Age Group			
0-39 years	N (%)	211917 (48.4%)	157731 (47.2%)
40-59 years	N (%)	123385 (28.2%)	93368 (28.0%)
60-79 years	N (%)	82315 (18.8%)	65559 (19.6%)
80+ years	N (%)	19973 (4.6%)	17336 (5.2%)
Number of Long-Term Conditions			
0	N (%)	163616 (37.4%)	151063 (45.2%)
1	N (%)	98470 (22.5%)	74497 (22.3%)
2	N (%)	63847 (14.6%)	43597 (13.1%)
3-4	N (%)	72814 (16.6%)	44006 (13.2%)
5+	N (%)	38843 (8.9%)	20831 (6.2%)

144 **Table 2** shows the mean number and duration of contacts between patients and clinical practice staff, by age, sex, and number of LTCs, and the estimated differences between practices serving a population living in relatively SE deprived areas, and practices serving more affluent areas. Confidence intervals are derived from simple two-sample t-tests, as an indication of which differences are important. **Figure 1** displays mean contact time by age, sex, and number of LTCs, for practices with SE deprived and affluent populations.

For peer review only - http://bmjopen.bmj.com/site/about/guidelines.xhtml

For peer review only

Table 2: Mean number and duration of appointments (over 3 years), by age, sex, and number of long-term conditions, for practices with SE deprived and affluent populations.

	Total Number of Appointments			Total Duration of Appointments (Minutes)		
	Practices in Deprived Areas	Practices in Affluent Areas	Difference (95% CI), p-value	Practices in Deprived Areas	Practices in Affluent Areas	Difference (95% CI), p-value
	Mean (SD)	Mean (SD)		Mean (SD)	Mean (SD)	
All	11.8 (14.6)	11.4 (14.2)	0.4 (0.3, 0.5), p<0.0001	146.1 (228.4)	149.5 (223.4)	-3.3 (-4.4, -2.3), p<0.0001
Sex						
Male	9.9 (13.2)	9.9 (13.3)	0.0 (-0.1, 0.1), p=0.4361	121.1 (207.0)	128.5 (209.6)	-7.4 (-8.8, -6.0), p<0.0001
Female	13.5 (15.6)	12.7 (14.8)	0.8 (0.7, 0.9), p<0.0001	168.4 (243.7)	167.7 (233.2)	0.7 (-0.8, 2.2), p=0.3576
Age						
0-39 years	8.2 (9.6)	7.6 (8.9)	0.5 (0.5, 0.6), p<0.0001	95.2 (135.1)	97.6 (143.4)	-2.4 (-3.3, -1.5), p<0.0001
40-59 years	12.7 (14.7)	11.3 (13.3)	1.3 (1.2, 1.5), p<0.0001	157.6 (222.6)	153.9 (223.4)	3.8 (1.9, 5.7), p=0.0001
60-79 years	17.6 (18.6)	17.3 (18.1)	0.3 (0.1, 0.5), p=0.0021	223.9 (299.5)	225.9 (284.8)	-2.0 (-5.0, 0.9), p=0.1802
80+ years	21.8 (24.1)	24.3 (23.5)	-2.5 (-3.0, -2.0), p<0.0001	294.5 (445.1)	308.1 (356.6)	-13.6 (-21.7, -5.4), p=0.0011
Number of Long-Term Conditions						
0	5.9 (6.7)	6.0 (6.5)	-0.1 (-0.2, -0.1), p<0.0001	66.9 (95.6)	74.7 (101.5)	-7.9 (-8.6, -7.2), p<0.0001
1	9.2 (9.4)	10.1 (9.9)	-0.8 (-0.9, -0.7), p<0.0001	107.0 (134.7)	130.8 (157.7)	-23.8 (-25.3, -22.4), p<0.0001
2	13.4 (13.0)	14.1 (13.1)	-0.7 (-0.8, -0.5), p<0.0001	161.1 (195.9)	187.9 (223.1)	-26.8 (-29.4, -24.2), p<0.0001
3-4	18.8 (17.5)	20.5 (18.4)	-1.7 (-1.9, -1.5), p<0.0001	236.8 (267.6)	275.0 (305.2)	-38.2 (-41.7, -34.8), p<0.0001
5+	27.7 (25.2)	30.7 (26.9)	-3.0 (-3.5, -2.6), p<0.0001	384.6 (450.3)	412.3 (434.1)	-27.7 (-35.1, -20.3), p<0.0001

For peer review only

For peer review only - http://bmjopen.bmj.com/site/about/guidelines.xhtml

Overall, patients in SE deprived areas had more discrete contacts with clinical practice staff over 3 years (11.8 vs.11.4), but each patient had less contact time (146.1 vs 149.5 minutes). Men in SE deprived areas had the same number of contacts, but spent 7.4 minutes less time with clinical staff, compared to men in affluent areas. Women in practices in SE deprived areas had slightly more contacts than women in affluent area practices (0.8 more contacts over 3 years); but spent a similar amount of time with clinical practice staff.

Patterns by age are more complex. For the youngest age group (under 40 years), and for those aged 60-70 years, patients in SE deprived area practices had marginally more contacts, but less time with clinical practice staff. In the 40-59 age band, however, patients in SE deprived areas had slightly more contacts, and spent slightly more time with clinical practice staff. In the oldest patients (over 80 years) patients registered to practices serving more SE deprived populations had fewer contacts and spent less time with the practice.

However, when looking at the data in relation to the number of LTCs that were recorded, there is a consistent and striking pattern. Patients in practices in SE deprived areas clearly have fewer contacts, and spend less time with clinical practice staff, compared to patients in more affluent practices, at every level of morbidity.

Table 3 shows how much contact time would be required between patients and staff in practices serving more SE deprived populations if the average contact time were the same as in practices with more affluent populations. Overall, practices in affluent areas spent about 50 million minutes with 334,000 patients, at an average of roughly 150 minutes per patient over the three-year study period. This was slightly more than the average contact time in practices in SE deprived areas, so that if these practices were to have the same average contact time, the total amount of time spent in contact with patients would need to increase by 2.3%.

For peer review only

For peer review only - http://bmjopen.bmj.com/site/about/guidelines.xhtml

Table 3: Additional resource requirements (in terms of total appointment time) for General Practices in deprived areas to offer equal appointment times to General Practices in affluent areas

		Practices in Affluent Areas (N=70)			Practices in Deprived Areas (N=80)				
		Observed Appointment Times (Minutes)			Observed Appointment Times (Minutes)			Assuming Equitable Appointment Times	
Stratification	Stratum	N Patients	Total	Mean	N Patients	Total	Mean	Total minutes	% Change Required
None		333994	49919411	149.5	437590	63942072	146.1	65403076	+2.3%
Sex	Male	155289	19951031	128.5	205914	24930987	121.1	26455168	+6.1%
	Female	178705	29968380	167.7	231676	39011085	168.4	38851484	-0.4%
	Overall							65306652	+2.1%
Age	0-39	157731	15401793	97.6	211917	20180456	95.2	20692836	+2.5%
	40-59	93368	14365312	153.9	123385	19450870	157.6	18983635	-2.4%
	60-79	65559	14811401	225.9	82315	18428732	223.9	18596996	+0.9%
	80+	17336	5340905	308.1	19973	5882014	294.5	6153317	+4.6%
	Overall							64426784	+0.8%
LTC Count	0	151063	11291456	74.7	163616	10942172	66.9	12229751	+11.8%
	1	74497	9744067	130.8	98470	10531390	107.0	12879690	+22.3%
	2	43597	8192478	187.9	63847	10285517	161.1	11997732	+16.6%
	3-4	44006	12103247	275.0	72814	17244417	236.8	20026492	+16.1%
	5+	20831	8588163	412.3	38843	14938576	384.6	16014114	+7.2%
	Overall							73147780	+14.4%
Age, Sex	Overall							64311097	+0.6%
Sex, LTC Count	Overall							72988341	+14.1%
Age, LTC Count	Overall							72834600	+13.9%
Sex, Age, LTC Count	Overall							72563329	+13.5%

For peer review only - http://bmjopen.bmj.com/site/about/guidelines.xhtml

The same calculations can be stratified by sex. The increase in contact time required in practices in SE deprived areas appears to be limited to male patients, but when the two sexes are combined, the adjustment required is similar to the overall calculation, at 2.1%. Broken down by age, different age bands are seen to require different adjustments, some positive and some negative, so that overall, the increase required in practices in SE deprived areas appears minimal, at 0.8%.

A different picture appears when stratifying the study population by number of LTCs. At every level of morbidity, patients in practices in SE deprived areas have less contact time with their practices, and practices would require a significant increase in resources to deliver the same level of care as practices in more SE affluent areas. The smallest increase required is 7.2%, for patients with the most complex needs, with five or more LTCs. When combined, these adjustments add up to an increase in contact time required in SE deprived areas of 14.4%.

Each of these calculations is stratified by only one condition, but the same calculations can use combinations of stratification factors, and these are shown in Table 3. Stratifying by age and sex alone, reveals that very little adjustment is required to achieve parity in terms of mean contact time. However, as soon as the number of LTCs are factored into the calculations, it is clear that a large increase in resources would be required for practices in SE deprived areas to offer the same amount of contact time to patients with the same level of need. Stratifying by age, sex, and number of LTCs, the estimated adjustment required is an increase of 13.5%.

Discussion

Summary

Using patient records from a sample of 150 Scottish general practices we demonstrate that substantially less care is received by patients in practices in socio-economically deprived areas compared to patients in affluent areas, but only when viewed in relation to morbidity, as measured by the number of LTCs. If general practices in highly SE deprived areas were to give an equivalent

For peer review only - http://bmjopen.bmj.com/site/about/guidelines.xhtml

amount of time (direct contact) to patients with the same level of need (the number of long-term conditions), they would require additional resources sufficient to support an increase of approximately 14% in patient contact time.

Strengths and limitations

This study utilises routine administrative data from a large population dataset which limits the potential bias associated with self-reported utilisation data[2] when seeking to measure unmet need. It demonstrates that data are retrievable from practice data systems to measure contact time with practice staff and allow for an estimation of resources required. The findings begin to address a universal problem about unmet need and resource allocation in primary care.

The accuracy of estimated time spent with patients has not been ascertained in research. However we have reduced any inaccuracies by using record opening times to best reflect clinical contacts.[11] Outside the Quality and Outcomes Framework,[14] there is also some variation in how long-term health conditions are coded. However, we followed established methods to mitigate for potential errors (6). We acknowledge that each long term condition included does not have equivalence in terms of impact on patients and that even within each condition there will be variation in the health care needs of individuals.[15]

The measure of socio-economic deprivation used is area-based, rather than one measured at an individual level to maximise accuracy; however this was the best available. Likewise we were unable – due to the anonymised nature of practice recruitment – to recruit a sample that was a perfect representation of the population; however our TTP recruited practices based on our specific request to ensure an urban/rural and affluent/SE deprived area spread.

Provision of medical care by general practices does have some distinct features depending on practice locality. For example, in remote rural settings practices provide more pre-hospital emergency care

For peer review only

For peer review only - http://bmjopen.bmj.com/site/about/guidelines.xhtml

which means more time may be spent with patients in those circumstances and this may bias consultation time estimates in these areas.

Finally these data predate the rapid and far reaching changes to general practice access in the UK triggered by the Covid pandemic. It will be some time yet before the longer-term consequences and eventual access arrangements settle into a new normal and their consequences are understood.

Comparison with existing literature

Research from Scotland over 20 years ago, and more recently, observed that consultations in more socio-economically deprived areas tended to be shorter[16, 17] and these findings have been replicated in England.[18] We know that provision of GP resource is flatly distributed in Scotland[19] and there are indications that the revised Scottish GP contract funding formula from 2018 has not had a positive impact in more SE deprived areas.[20] An in-depth analysis conducted with English routine administrative data showed that practices in high SE deprivation areas have fewer GPs and more practice nurses than affluent area-based counterparts. This means GPs in those settings are caring for 10% more patients overall.[21] What this paper demonstrates, for the first time, is how clinically focussed evidence that relies on high quality data from a large percentage of the Scottish population can inform and improve models of care. Put simply, a patient registered at a practice serving an affluent population will receive more care from their practice than a patient of the same age, sex, and level of morbidity, registered with a practice serving a more SE deprived population.

Implications for research and/or practice

This study adds to growing evidence that provision of general practice care in the UK is not equitably distributed across practices and needs to change.[22] Patients with long-term health conditions living in the least affluent areas are losing out. This is unacceptable unmet need because it is systematically related to socio-economic factors.

For peer review only - http://bmjopen.bmj.com/site/about/guidelines.xhtml

We estimate a 14% increase in clinical staff contact time is required in SE deprived areas to equalise provision. A direct impact of this analysis is that governments, health service policy makers and planners should consider using data on contact time to measure the amount of care delivered to patients, as well as data on the number of recorded LTCs to estimate the level of need of GP practice populations. This is now achievable through routine administrative data capture and should be factored into resource allocation algorithms. This paper adds to the large body of work now accumulated in the UK about the role general practice care has in perpetuating health inequalities and the inverse care law.[23]

Conclusion

General practice provision in SE deprived areas needs to be better supported to deliver uniform care to patients with the same level of need across the UK. The analysis from this paper suggests that measuring LTCs and funding accordingly would make a significant contribution to achieving this aim.

Ethics approval

Letters of comfort were issued by the West of Scotland NHS Ethics Committee and the University of Glasgow College of Medical, Veterinary & Life Sciences Ethics Committee confirming that the full study did not need NHS ethics approval as this used routine data that had been extracted in an anonymised format. Public Benefit and Privacy Panel approval for use of the data was granted by NHS Information Services Scotland in December 2016.

Author contributions

AEW: Designed and managed the study, wrote first draft of manuscript, reviewed final version of manuscript. DE, PW: Designed and managed the study, reviewed final version of manuscript. RM: Processed data for analysis, reviewed final version of manuscript. AM: Conceived and carried out statistical analysis, wrote statistical methods and results, reviewed final version of manuscript.

For peer review only - http://bmjopen.bmj.com/site/about/guidelines.xhtml

Data Availability

These data were available from NHS Scotland and access permissions were contingent on them not being publicly available. New data access and TTP requests can be made to www.escro.co.uk for general practice data and https://www.isdscotland.org/products-and-services/edris/ to host the data securely and request data linkage. The authors had no special access permissions that others could not have. Analysis code is available from the authors upon reasonable request. The code will replicate the study findings, but the analysis can be replicated using other statistical software.

Acknowledgements

Thank you to all the GP practices who participated in the Serial Missed Appointments study and for strategic support from Ellen Lynch (Health and Social Care Analytical Services, Scottish Government). The general practice data expertise of Dave Kelly (Albasoft) was invaluable. Thanks also to the eDRIS team who facilitated the safe use of our data in the Safehaven, especially Dionysis Vragkos.

Patient and Public Involvement

The Royal College of General Practitioners Scotland Patient Participation in Practice (P3) Committee in 2016 provided advice about the relevance and importance of this research. This was to inform the study team's application for data linkage of routine data sets. The P3 committee advised they agreed it was 'interesting, much needed and beneficial' and they have been updated on progress as the project has proceeded.

Figure Legends

Figure 1: Mean duration of appointments (over 3 years), by age, sex, and number of long-term conditions, for practices with SE deprived and affluent populations.

For peer review only

For peer review only - http://bmjopen.bmj.com/site/about/guidelines.xhtml

References

1. Smith S, Connolly S. Re-thinking unmet need for health care: introducing a dynamic perspective. *Health Econ Policy Law*. 2020;15(4):440-57.

2. Allin S, Grignon M, Le Grand J. Subjective unmet need and utilization of health care services in Canada: What are the equity implications? *Soc Sci Med*. 2010;70(3):465-72.

3. Pearson-Stuttard J, Ezzati M, Gregg EW. Multimorbidity-a defining challenge for health systems. *Lancet Public Health*. 2019;4(12):e599-e600.

4. Whitty CJM, MacEwen C, Goddard A, et al. Rising to the challenge of multimorbidity. *BMJ*. 2020;368:l6964.

5. Rolewicz L, Keeble E, Paddison C, et al. Are the needs of people with multiple long-term conditions being met? Evidence from the 2018 General Practice Patient Survey. *BMJ Open*. 2020;10(11):e041569.

6. Barnett K, Mercer SMN, Watt G, et al. Epidemiology of Multi-morbidity and implications for health care, research and medical education: a cross sectional study. *Lancet*. 2012;380((9836)):37-43.

7. Menotti A, Mulder I, Nissinen A, et al. Prevalence of morbidity and multimorbidity in elderly male populations and their impact on 10-year all-cause mortality: The FINE study (Finland, Italy, Netherlands, Elderly). *J Clin Epidemiol*. 2001;54(7):680-6.

8. Stanley J, Semper K, Millar E, et al. Epidemiology of multimorbidity in New Zealand: a cross-sectional study using national-level hospital and pharmaceutical data. *BMJ Open*. 2018;8(5):e021689.

9. Albasoft, role and responsibilities as an NHS Trusted Third Party [Available from: http://www.escro.co.uk/marketing/TTP%20in%20primary%20care%20summary.pdf.

10. Ellis DA, McQueenie R, McConnachie A, et al. Demographic and practice factors predicting repeated non-attendance in primary care: a national retrospective cohort analysis. *Lancet Public Health*. 2017;2.

11. Williamson AE, Ellis DA, Wilson P, et al. Understanding repeated non-attendance in health services: a pilot analysis of administrative data and full study protocol for a national retrospective cohort. *BMJ Open*. 2017;7.

12. McQueenie R, Ellis DA, McConnachie A, et al. Morbidity, mortality and missed appointments in healthcare: a national retrospective data linkage study. *BMC Med*. 2019;17(1):2.

13. Scottish Index of Multiple Deprivation 2012 [Internet]. Scottish Government. 2012. Available from: http://www.gov.scot/Topics/Statistics/SIMD/DataAnalysis/Background-Data-2012.

14. Information Services Division NHS Scotland. General Practice Quality and Outcomes Framework UK: Public Health Scotland; 2020 [Available from: https://www.isdscotland.org/health-topics/general-practice/quality-and-outcomes-framework/.

15. Johnston MC, Crilly M, Black C, et al. Defining and measuring multimorbidity: a systematic review of systematic reviews. *Eur J Public Health*. 2019;29(1):182-9.

16. Stirling AM, Wilson P, McConnachie A. Deprivation, psychological distress, and consultation length in general practice. *Br J Gen Pract*. 2001;51(467):456-60.

17. Mercer SW, Watt GCM. The inverse care law: clinical primary care encounters in deprived and affluent areas of Scotland. *Ann Fam Med*. 2007;5(6):503-10.

18. Gopfert A, Deeny SR, Fisher R, et al. Primary care consultation length by deprivation and multimorbidity in England: an observational study using electronic patient records. *Br J Gen Pract*. 2021;71(704):e185-e92.

19. McLean G, Guthrie B, Mercer SW, et al. General practice funding underpins the persistence of the inverse care law: cross-sectional study in Scotland. *Br J Gen Pract*. 2015;65(641):e799-e805.

20. Morton C. Health Inequalities in Scotland A National Calamity; a Frontline GP view. UK: Independently published; 2020.

For peer review only

For peer review only - http://bmjopen.bmj.com/site/about/guidelines.xhtml

21. Fisher R, Dunn P, Asaria M, et al. Level or not? Comparing general practice in areas of high and low socioeconomic deprivation in England UK; 2020.

22. Ashworth M, L'Esperance V, Round T. Primary care funding entrenches health inequalities: time for a rethink. *Br J Gen Pract*. 2021;71(704):102-4.

23. Cookson R, Doran T, Asaria M, et al. The inverse care law re-examined: a global perspective. *Lancet*. 2021;397(10276):828-38.

For peer review only - http://bmjopen.bmj.com/site/about/guidelines.xhtml

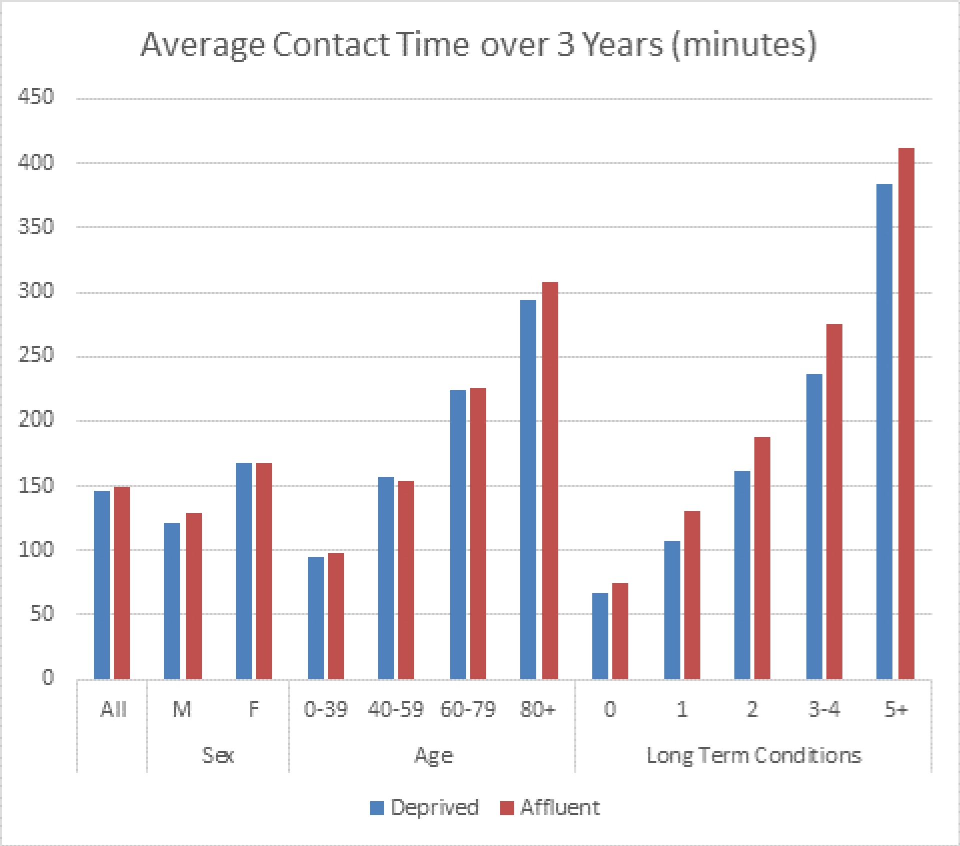

127x111mm (96 x 96 DPI)

The RECORD statement – checklist of items, extended from the STROBE statement, that should be reported in observational studies using routinely collected health data.

	Item No.	STROBE items	Location in manuscript where items are reported	RECORD items	Location in manuscript where items are reported
**Title and abstract**					
	1	(a) Indicate the study's design with a commonly used term in the title or the abstract<br><br>(b) Provide in the abstract an informative and balanced summary of what was done and what was found	Title<br><br>p2, line 23-45	RECORD 1.1: The type of data used should be specified in the title or abstract. When possible, the name of the databases used should be included.<br><br>RECORD 1.2: If applicable, the geographic region and timeframe within which the study took place should be reported in the title or abstract.<br><br>RECORD 1.3: If linkage between databases was conducted for the study, this should be clearly stated in the title or abstract.	Title<br><br>p2, line 29-31<br><br>N/A
**Introduction**					
Background rationale	2	Explain the scientific background and rationale for the investigation being reported			p4, line 73-93
Objectives	3	State specific objectives, including any prespecified hypotheses			p4, line 91-93
**Methods**					
Study Design	4	Present key elements of study design early in the paper			Title<br>p5, line 96-107
Setting	5	Describe the setting, locations, and relevant dates, including periods of recruitment, exposure, follow-up, and data collection			p5, line 96-107

For peer review only

Participants	6	*(a) Cohort study* - Give the eligibility criteria, and the sources and methods of selection of participants. Describe methods of follow-up<br>*Case-control study* - Give the eligibility criteria, and the sources and methods of case ascertainment and control selection. Give the rationale for the choice of cases and controls<br>*Cross-sectional study* - Give the eligibility criteria, and the sources and methods of selection of participants<br><br>*(b) Cohort study* - For matched studies, give matching criteria and number of exposed and unexposed<br>*Case-control study* - For matched studies, give matching criteria and the number of controls per case	p5, line 96-114<br><br>N/A	RECORD 6.1: The methods of study population selection (such as codes or algorithms used to identify subjects) should be listed in detail. If this is not possible, an explanation should be provided.<br><br>RECORD 6.2: Any validation studies of the codes or algorithms used to select the population should be referenced. If validation was conducted for this study and not published elsewhere, detailed methods and results should be provided.<br><br>RECORD 6.3: If the study involved linkage of databases, consider use of a flow diagram or other graphical display to demonstrate the data linkage process, including the number of individuals with linked data at each stage.	p5, line 102-106<br><br>N/A<br><br>N/A
Variables	7	Clearly define all outcomes, exposures, predictors, potential confounders, and effect modifiers. Give diagnostic criteria, if applicable.	p5-6, line 115-124	RECORD 7.1: A complete list of codes and algorithms used to classify exposures, outcomes, confounders, and effect modifiers should be provided. If these cannot be reported, an explanation should be provided.	p15, line 269-274
Data sources/ measurement	8	For each variable of interest, give sources of data and details of methods of assessment (measurement).<br>Describe comparability of assessment methods if there is more than one group			p5-6, line 96-124

For peer review only

Bias	9	Describe any efforts to address potential sources of bias			p12-13, line 203-223
Study size	10	Explain how the study size was arrived at			N/A
Quantitative variables	11	Explain how quantitative variables were handled in the analyses. If applicable, describe which groupings were chosen, and why			p5, line 109-114
Statistical methods	12	(a) Describe all statistical methods, including those used to control for confounding<br>(b) Describe any methods used to examine subgroups and interactions<br>(c) Explain how missing data were addressed<br>(d) *Cohort study* - If applicable, explain how loss to follow-up was addressed<br>*Case-control study* - If applicable, explain how matching of cases and controls was addressed<br>*Cross-sectional study* - If applicable, describe analytical methods taking account of sampling strategy<br>(e) Describe any sensitivity analyses			p6, line 125-130
Data access and cleaning methods		..		RECORD 12.1: Authors should describe the extent to which the investigators had access to the database population used to create the study population.	p5, line 108

For peer review only - http://bmjopen.bmj.com/site/about/guidelines.xhtml

				RECORD 12.2: Authors should provide information on the data cleaning methods used in the study.	p5, line 108
Linkage		..		RECORD 12.3: State whether the study included person-level, institutional-level, or other data linkage across two or more databases. The methods of linkage and methods of linkage quality evaluation should be provided.	N/A
**Results**					
Participants	13	(a) Report the numbers of individuals at each stage of the study (*e.g.*, numbers potentially eligible, examined for eligibility, confirmed eligible, included in the study, completing follow-up, and analysed)<br>(b) Give reasons for non-participation at each stage.<br>(c) Consider use of a flow diagram	p6, line 133-137	RECORD 13.1: Describe in detail the selection of the persons included in the study (*i.e.,* study population selection) including filtering based on data quality, data availability and linkage. The selection of included persons can be described in the text and/or by means of the study flow diagram.	p6, line 133-137
Descriptive data	14	(a) Give characteristics of study participants (*e.g.*, demographic, clinical, social) and information on exposures and potential confounders<br>(b) Indicate the number of participants with missing data for each variable of interest<br>(c) *Cohort study* - summarise follow-up time (*e.g.*, average and total amount)			p6-7, line 137-143
Outcome data	15	*Cohort study* - Report numbers of outcome events or summary measures over time<br>*Case-control study* - Report numbers in each exposure			p7-9, line 144-166

For peer review only

		category, or summary measures of exposure<br>*Cross-sectional study* - Report numbers of outcome events or summary measures			
Main results	16	(a) Give unadjusted estimates and, if applicable, confounder-adjusted estimates and their precision (e.g., 95% confidence interval). Make clear which confounders were adjusted for and why they were included<br>(b) Report category boundaries when continuous variables were categorized<br>(c) If relevant, consider translating estimates of relative risk into absolute risk for a meaningful time period			p9-11, line 167-192
Other analyses	17	Report other analyses done—e.g., analyses of subgroups and interactions, and sensitivity analyses			p9-11, line 167-192
**Discussion**					
Key results	18	Summarise key results with reference to study objectives			p11-12, line 195-201
Limitations	19	Discuss limitations of the study, taking into account sources of potential bias or imprecision. Discuss both direction and magnitude of any potential bias	p12-13, line 203-226	RECORD 19.1: Discuss the implications of using data that were not created or collected to answer the specific research question(s). Include discussion of misclassification bias, unmeasured confounding, missing data, and changing eligibility over time, as they pertain to the study being reported.	p12-13, line 203-226
Interpretation	20	Give a cautious overall interpretation of results			p13-14, line 241-252

For peer review only

		considering objectives, limitations, multiplicity of analyses, results from similar studies, and other relevant evidence			
Generalisability	21	Discuss the generalisability (external validity) of the study results			p12-13, line 215-223
**Other Information**					
Funding	22	Give the source of funding and the role of the funders for the present study and, if applicable, for the original study on which the present article is based			p3, line 64-68
Accessibility of protocol, raw data, and programming code		..		RECORD 22.1: Authors should provide information on how to access any supplemental information such as the study protocol, raw data, or programming code.	p3, line 62<br>p15, line 269-274

*Reference: Benchimol EI, Smeeth L, Guttmann A, Harron K, Moher D, Petersen I, Sørensen HT, von Elm E, Langan SM, the RECORD Working Committee. The REporting of studies Conducted using Observational Routinely-collected health Data (RECORD) Statement. *PLoS Medicine* 2015; in press.

*Checklist is protected under Creative Commons Attribution (CC BY) license.

For peer review only

BMJ Open

Quantifying unmet need in General Practice: a retrospective cohort study of administrative data

Journal:	*BMJ Open*
Manuscript ID	bmjopen-2022-068720.R1
Article Type:	Original research
Date Submitted by the Author:	23-Jan-2023
Complete List of Authors:	McConnachie, Alex; University of Glasgow, Robertson Centre for Biostatistics<br>Ellis, David; University of Bath, School of Management<br>Wilson, Philip; University of Aberdeen, Institute of Health and Wellbeing<br>McQueenie, Ross; University of Glasgow, Genera Practice and Primary Care<br>Williamson, Andrea; University of Glasgow, GPPC, School of Medicine, Dentistry and Nursing, MVLS
**Primary Subject Heading**:	General practice / Family practice
Secondary Subject Heading:	Health services research
Keywords:	PRIMARY CARE, Organisation of health services < HEALTH SERVICES ADMINISTRATION & MANAGEMENT, Quality in health care < HEALTH SERVICES ADMINISTRATION & MANAGEMENT

SCHOLARONE™
Manuscripts

I, the Submitting Author has the right to grant and does grant on behalf of all authors of the Work (as defined in the below author licence), an exclusive licence and/or a non-exclusive licence for contributions from authors who are: i) UK Crown employees; ii) where BMJ has agreed a CC-BY licence shall apply, and/or iii) in accordance with the terms applicable for US Federal Government officers or employees acting as part of their official duties; on a worldwide, perpetual, irrevocable, royalty-free basis to BMJ Publishing Group Ltd ("BMJ") its licensees and where the relevant Journal is co-owned by BMJ to the co-owners of the Journal, to publish the Work in this journal and any other BMJ products and to exploit all rights, as set out in our licence.

The Submitting Author accepts and understands that any supply made under these terms is made by BMJ to the Submitting Author unless you are acting as an employee on behalf of your employer or a postgraduate student of an affiliated institution which is paying any applicable article publishing charge ("APC") for Open Access articles. Where the Submitting Author wishes to make the Work available on an Open Access basis (and intends to pay the relevant APC), the terms of reuse of such Open Access shall be governed by a Creative Commons licence – details of these licences and which Creative Commons licence will apply to this Work are set out in our licence referred to above.

Other than as permitted in any relevant BMJ Author's Self Archiving Policies, I confirm this Work has not been accepted for publication elsewhere, is not being considered for publication elsewhere and does not duplicate material already published. I confirm all authors consent to publication of this Work and authorise the granting of this licence.

For peer review only

Quantifying unmet need in General Practice: a retrospective cohort study of administrative data

Alex McConnachie[1], David A Ellis[2], Philip Wilson[3], Ross McQueenie[4], Andrea E Williamson[5]

[1]BSc MSc PhD, Professor of Clinical Trial Biostatistics, Robertson Centre for Biostatistics, School of Health and Wellbeing, MVLS, University of Glasgow, Scotland, UK. ORCID ID: 0000-0002-7262-7000

[2] MA MSc PhD, Associate Professor in Information Systems; School of Management, University of Bath, Claverton Down, Bath BA2 7AY, UK. ORCID ID: 0000-0001-6172-3323

[3] MA MB BChir DPhil DCH FRCPCH FRCGP, Professor emeritus, Institute of Applied Health Sciences, University of Aberdeen, Scotland, UK. ORCID ID: 0000-0002-4123-8248

[4] BSc Hons, PhD, Research Associate, General Practice and Primary Care, School of Health and Wellbeing, MVLS, University of Glasgow, 1 Horselethill Road, Glasgow, G12 9LX UK. ORCID ID: 0000-0002-0070-6607

[5] MBChB MPH PhD FRCGP FHEA DTM&H, Professor of General Practice and Inclusion Health, General Practice and Primary Care, School of Health and Wellbeing, MVLS, University of Glasgow, 1 Horselethill Road, Glasgow, G12 9LX UK. ORCID ID: 0000-0002-8981-9068 @aewilliamsonl

Correspondence to: Professor Alex McConnachie, Robertson Centre for Biostatistics, School of Health and Wellbeing, MVLS, University of Glasgow, Scotland, UK, alex.mcconnachie@glasgow.ac.uk

Word count (excluding abstract, references, tables, figures): 3090

For peer review only

For peer review only - http://bmjopen.bmj.com/site/about/guidelines.xhtml

Abstract

Objectives: To assess whether patients attending General Practices (GPs) in socioeconomically deprived areas receive the same level of care, compared to similar patients (based on age, sex, and level of morbidity) attending GPs in less deprived areas. If not, to quantify the additional resource that would be required by GPs in deprived areas to achieve parity.

Design: Retrospective cohort study.

Setting: 150 General Practices in Scotland, UK, divided into two groups: 80 practices in Scottish Index of Multiple Deprivation (SIMD) decile 1-5 (more SE deprived); 70 practices in SIMD decile 6-10 (less SE deprived).

Patients: 437,590 patients registered with a more SE deprived GP, and 333,994 patients registered with a less SE deprived GP, for the whole study period (2013-2016), who made at least one appointment.

Outcomes: The number of contacts and total contact time between patients and clinical staff.

Results: Patients in more SE deprived areas had slightly more discrete contacts over 3 years (11.8 vs. 11.4), but each patient had marginally less contact time (146.1 vs 149.5 minutes). Stratified by sex and age, differences were also small. Stratified by the number of Long-Term Conditions (nLTCs), practices in more SE deprived areas delivered significantly less contact time than practices in less SE deprived areas. Over three years, 8 fewer minutes for patients with no LTCs, and 24, 27, 38, and 28 fewer minutes for patients with 1, 2, 3-4, or 5+ LTCs, respectively.

Conclusion: If GPs in more SE deprived areas were to give an equal amount of direct contact time to patients with the same level of need served by GPs in less SE deprived areas, this would require a 14% increase in patient contact time. This represents a significant unmet need, supporting the case for redistribution of resources to tackle the inverse care law.

Keywords: general practice, health care planning, unmet need, inverse care law, inequalities, big data

For peer review only - http://bmjopen.bmj.com/site/about/guidelines.xhtml

Strengths and limitations

Strengths

- Large dataset, based on routine administrative records, demonstrating the feasibility of measuring contact time with practice staff.
- Large sample of practices covering urban and rural, affluent and deprived areas.

Limitations

- The accuracy of time spent with patients based on the opening and closing of medical records has not been verified.
- Health care need is estimated by a simple count of the number of long-term conditions, which does not reflect variation in health care needs between conditions, nor variation in needs between patients within the same condition.
- These data predate the rapid and far reaching changes to general practice access in the UK triggered by the Covid pandemic.

For peer review only

For peer review only - http://bmjopen.bmj.com/site/about/guidelines.xhtml

Introduction

Unmet need is a multidimensional, complex construct;[1] "*when an individual does not receive an available and effective treatment that could have improved her health…some unmet need is acceptable since resources are scarce. What is of concern here is whether unmet need is inequitable, or systematically related to socioeconomic or other personal characteristics*".[2]

Patients living with multi-morbidity (two or more co-existing long-term health conditions (LTCs)) have complex needs, and successfully meeting those within our healthcare systems is one of the most pressing challenges of our time.[3-5] We already have strong evidence that people living in more socio-economically (SE) deprived areas in the UK have a burden of multi-morbidity at an earlier age.[6] Furthermore, patients with multi-morbidity are at greater risk of all-cause mortality[7, 8] and hospitalisation,[8] regardless of whether the number of LTCs are measured in a research setting or via primary care records,[9] suggesting that the numbers of LTCs recorded in general practice electronic health records broadly reflect underlying healthcare need.

The analyses presented in this paper stem from research exploring the determinants and outcomes of missed appointments in general practice in Scotland. During this work, we determined the total number and duration of contacts between clinical practice staff and individual patients. We postulated that patients with more LTCs have greater healthcare need, and therefore require more contact time with clinical practice staff. In an equitable health service, equivalent patients (according to their age, sex, and number of LTCs) should, on average, receive the same level of contact with clinical practice staff, regardless of whether they live in an affluent or socio-economically (SE) deprived area. Therefore, we hypothesised that patients in SE deprived communities may receive less clinical GP staff contact time with an equivalent level of morbidity. If this is the case, how much more contact time would be required in socio-economically deprived practices to achieve parity with affluent practices?

For peer review only - http://bmjopen.bmj.com/site/about/guidelines.xhtml

Methods

A Trusted Third party (TTP) for the National Health Service (NHS) in Scotland (Albasoft), recruited general practices (GPs) on our behalf. A TTP is an organisation independent from the NHS with the skills, infrastructure and permissions to extract and link data safely without having a direct interest in the use to which the data will be put. The TTP is required to ensure confidentiality for patients and professionals in the research process.[10] The TTP recruited practices to ensure a spread of practices in urban and rural locations, serving both affluent and socio-economically (SE) deprived (Deep End) populations. All patients who were alive and registered at the same practice for the 3-year period (5th September 2013 to 5th September 2016) and who made at least one appointment with the practice were included in the data set. All patients retained also had data on age, sex, number of recorded LTCs, and the number and total duration of all in person contacts with clinical practice staff such as GPs and practice nurses. Recording of patient ethnicity and language interpreter requirements was very low so this could not be included in our analysis.[11]

How we defined and measured LTCs are documented in previous publications.[11-13]

We present the number and percentage of patients in practices serving affluent and deprived populations, according to sex, age (0-39, 40-59, 60-79, and 80+ years), and the number of LTCs (none, 1, 2, 3-4, or 5+). Practices were categorised as affluent or deprived based on the average Scottish Index of Multiple Deprivation (SIMD) score of all patients registered at the practice. SIMD is routinely used in Scotland as part of the current GP contract funding allocation formula. When split by decile groups, 1 is the most SE deprived.[14]

We calculated the total number and duration of contacts in minutes for each patient. These were extracted from appointments recorded via practice IT systems. The duration of contacts was calculated from the 'in' and 'out' time. Detailed proof of concept work in our pilot study of 67,705 patient records enabled us to set rules about included appointments to increase accuracy.

For peer review only - http://bmjopen.bmj.com/site/about/guidelines.xhtml

Appointments that were 'open' for less than 2 minutes, 'open' for more than 24 hours, labelled as an administrative slot, or were not face-to-face appointments were excluded.[12] There is no unified categorisation of General Practice appointment types in the UK and this was reflected in the heterogeneity of appointment categories in our data set. Therefore, all appointments included were for face-to-face appointments with a health care professional in the practice (rather than being specific e.g. about GP or practice nurse contacts).[11]

We present the mean and standard deviation (SD) of the number and duration of contacts for patients in practices in affluent and deprived areas, according to sex, age, and the number of LTCs.

Taking the mean contact time provided by practices in affluent areas and applying this to the number of patients served by practices in SE deprived areas, we calculated the change in total contact time that would be required in practices in SE deprived areas in order to give the same average contact time. We then repeated this analysis, stratified by sex, age, and/or number of LTCs.

All analyses were done using the R statistical software package [15].

Patient and Public Involvement

The Royal College of General Practitioners Scotland Patient Partnership in Practice (P^3) Committee in 2016 provided advice about the relevance and importance of this research. This was to inform the study team's application for data linkage of routine data sets. The P^3 committee (a lay patient group) advised that they agreed it was 'interesting, much needed and beneficial' and they have been updated on progress as the project has proceeded.

Results

Data from 150 Scottish practices were included. These were split into two groups: SIMD decile 1-5 (most SE deprived, 80 practices) and SIMD decile 6-10 (least deprived, 70 practices). From a cohort of 824,374 patients, after exclusion of patients who moved practice, or who had missing data for age,

For peer review only

For peer review only - http://bmjopen.bmj.com/site/about/guidelines.xhtml

sex, or number of LTCs, data from 771,584 patient records were analysed. The study population was 437,590 patients from the 80 practices in SE deprived areas, and 333,994 patients from the 70 practices in affluent areas. **Table 1** sets out their characteristics. Differences in terms of the sex and age distributions are small. 47% of patients from SE deprived practices in this sample were male, compared to 46% of patients in practices in more affluent areas. Patients registered with practices in SE deprived areas were slightly younger, with 48% vs. 47% being under 40, and 4.6% vs. 5.2% being over 80 years of age. The difference in terms of the distribution of the number of LTCs that patients had was far more marked, with 45% of patients in practices serving affluent populations having no LTCs, compared to 37% of patients in practices serving SE deprived populations.

Table 1: Study population. Numbers and percentages of patients in groups defined by sex, age, and number of long-term conditions, for practices with SE deprived and affluent populations.

		Practices in Deprived Areas	Practices in Affluent Areas
Number of Practices		80	70
Number of Patients		437,590	333,994
Sex			
Male	N (%)	205,914 (47.1%)	155,289 (46.5%)
Female	N (%)	231,676 (52.9%)	178,705 (53.5%)
Age Group			
0-39 years	N (%)	211,917 (48.4%)	157,731 (47.2%)
40-59 years	N (%)	123,385 (28.2%)	93,368 (28.0%)
60-79 years	N (%)	82,315 (18.8%)	65,559 (19.6%)
80+ years	N (%)	19,973 (4.6%)	17,336 (5.2%)
Number of Long-Term Conditions			
0	N (%)	163,616 (37.4%)	151,063 (45.2%)
1	N (%)	98,470 (22.5%)	74,497 (22.3%)
2	N (%)	63,847 (14.6%)	43,597 (13.1%)
3-4	N (%)	72,814 (16.6%)	44,006 (13.2%)
5+	N (%)	38,843 (8.9%)	20,831 (6.2%)

Table 2 shows the mean number and duration of contacts between patients and clinical practice staff, by age, sex, and number of LTCs, and the estimated differences between practices serving a

For peer review only - http://bmjopen.bmj.com/site/about/guidelines.xhtml

For peer review only

population living in relatively SE deprived areas, and practices serving more affluent areas. Confidence intervals are derived from simple two-sample t-tests. **Figure 1** displays mean contact time by age, sex, and number of LTCs, for practices with SE deprived and affluent populations.

For peer review only

For peer review only - http://bmjopen.bmj.com/site/about/guidelines.xhtml

Table 2: Mean number and duration of appointments (over 3 years), by age, sex, and number of long-term conditions, for practices with SE deprived and affluent populations.

	Total Number of Appointments			Total Duration of Appointments (Minutes)		
	Practices in Deprived Areas	Practices in Affluent Areas	Difference (95% CI), p-value	Practices in Deprived Areas	Practices in Affluent Areas	Difference (95% CI), p-value
	Mean (SD)	Mean (SD)		Mean (SD)	Mean (SD)	
All	11.8 (14.6)	11.4 (14.2)	0.4 (0.3, 0.5), p<0.0001	146.1 (228.4)	149.5 (223.4)	-3.3 (-4.4, -2.3), p<0.0001
Sex						
Male	9.9 (13.2)	9.9 (13.3)	0.0 (-0.1, 0.1), p=0.4361	121.1 (207.0)	128.5 (209.6)	-7.4 (-8.8, -6.0), p<0.0001
Female	13.5 (15.6)	12.7 (14.8)	0.8 (0.7, 0.9), p<0.0001	168.4 (243.7)	167.7 (233.2)	0.7 (-0.8, 2.2), p=0.36
Age						
0-39 years	8.2 (9.6)	7.6 (8.9)	0.5 (0.5, 0.6), p<0.0001	95.2 (135.1)	97.6 (143.4)	-2.4 (-3.3, -1.5), p<0.0001
40-59 years	12.7 (14.7)	11.3 (13.3)	1.3 (1.2, 1.5), p<0.0001	157.6 (222.6)	153.9 (223.4)	3.8 (1.9, 5.7), p=0.0001
60-79 years	17.6 (18.6)	17.3 (18.1)	0.3 (0.1, 0.5), p=0.0021	223.9 (299.5)	225.9 (284.8)	-2.0 (-5.0, 0.9), p=0.18
80+ years	21.8 (24.1)	24.3 (23.5)	-2.5 (-3.0, -2.0), p<0.0001	294.5 (445.1)	308.1 (356.6)	-13.6 (-21.7, -5.4), p=0.0011
Number of Long-Term Conditions						
0	5.9 (6.7)	6.0 (6.5)	-0.1 (-0.2, -0.1), p<0.0001	66.9 (95.6)	74.7 (101.5)	-7.9 (-8.6, -7.2), p<0.0001
1	9.2 (9.4)	10.1 (9.9)	-0.8 (-0.9, -0.7), p<0.0001	107.0 (134.7)	130.8 (157.7)	-23.8 (-25.3, -22.4), p<0.0001
2	13.4 (13.0)	14.1 (13.1)	-0.7 (-0.8, -0.5), p<0.0001	161.1 (195.9)	187.9 (223.1)	-26.8 (-29.4, -24.2), p<0.0001
3-4	18.8 (17.5)	20.5 (18.4)	-1.7 (-1.9, -1.5), p<0.0001	236.8 (267.6)	275.0 (305.2)	-38.2 (-41.7, -34.8), p<0.0001
5+	27.7 (25.2)	30.7 (26.9)	-3.0 (-3.5, -2.6), p<0.0001	384.6 (450.3)	412.3 (434.1)	-27.7 (-35.1, -20.3), p<0.0001

For peer review only

For peer review only - http://bmjopen.bmj.com/site/about/guidelines.xhtml

Overall, patients in SE deprived areas had more discrete contacts with clinical practice staff over 3 years (11.8 vs.11.4), but each patient had less contact time (146.1 vs 149.5 minutes). Men in SE deprived areas had the same number of contacts, but spent 7.4 minutes less time with clinical staff, compared to men in affluent areas. Women in practices in SE deprived areas had slightly more contacts than women in affluent area practices (0.8 more contacts over 3 years); but spent a similar amount of time with clinical practice staff.

Patterns by age are more complex. For the youngest age group (under 40 years), and for those aged 60-70 years, patients in SE deprived area practices had marginally more contacts, but less time with clinical practice staff. In the 40-59 age band, however, patients in SE deprived areas had slightly more contacts, and spent slightly more time with clinical practice staff. In the oldest patients (over 80 years) patients registered to practices serving more SE deprived populations had fewer contacts and spent less time with the practice.

However, when looking at the data in relation to the number of LTCs that were recorded, there is a consistent and striking pattern. Patients in practices in SE deprived areas clearly have fewer contacts, and spend less time with clinical practice staff, compared to patients in more affluent practices, at every level of morbidity.

Table 3 shows how much contact time would be required between patients and staff in practices serving more SE deprived populations if the average contact time were the same as in practices with more affluent populations. Overall, practices in affluent areas spent about 50 million minutes with 334,000 patients, at an average of roughly 150 minutes per patient over the three-year study period. This was slightly more than the average contact time in practices in SE deprived areas, so that if these practices were to have the same average contact time, the total amount of time spent in contact with patients would need to increase by 2.3%.

For peer review only - http://bmjopen.bmj.com/site/about/guidelines.xhtml

Table 3: Additional resource requirements (in terms of total appointment time) for General Practices in deprived areas to offer equal appointment times to General Practices in affluent areas

		Practices in Affluent Areas (N=70)			Practices in Deprived Areas (N=80)				
		Observed Appointment Times (Minutes)			Observed Appointment Times (Minutes)			Assuming Equitable Appointment Times	
Stratification	Stratum	N Patients	Total	Mean	N Patients	Total	Mean	Total minutes	% Change Required
None		333,994	49,919,411	149.5	437,590	63,942,072	146.1	65,403,076	+2.3%
Sex	Male	155,289	19,951,031	128.5	205,914	24,930,987	121.1	26,455,168	+6.1%
	Female	178,705	29,968,380	167.7	231,676	39,011,085	168.4	38,851,484	-0.4%
	Overall							65,306,652	+2.1%
Age	0-39	157,731	15,401,793	97.6	211,917	20,180,456	95.2	20,692,836	+2.5%
	40-59	93,368	14,365,312	153.9	123,385	19,450,870	157.6	18,983,635	-2.4%
	60-79	65,559	14,811,401	225.9	82,315	18,428,732	223.9	18,596,996	+0.9%
	80+	17,336	5,340,905	308.1	19,973	5,882,014	294.5	6,153,317	+4.6%
	Overall							64,426,784	+0.8%
LTC Count	0	151,063	11,291,456	74.7	163,616	10,942,172	66.9	12,229,751	+11.8%
	1	74,497	9,744,067	130.8	98,470	10,531,390	107.0	12,879,690	+22.3%
	2	43,597	8,192,478	187.9	63,847	10,285,517	161.1	11,997,732	+16.6%
	3-4	44,006	12,103,247	275.0	72,814	17,244,417	236.8	20,026,492	+16.1%
	5+	20,831	8,588,163	412.3	38,843	14,938,576	384.6	16,014,114	+7.2%
	Overall							73,147,780	+14.4%
Age, Sex	Overall							64,311,097	+0.6%
Sex, LTC Count	Overall							72,988,341	+14.1%
Age, LTC Count	Overall							72,834,600	+13.9%
Sex, Age, LTC Count	Overall							72,563,329	+13.5%

For peer review only - http://bmjopen.bmj.com/site/about/guidelines.xhtml

The same calculations can be stratified by sex. The increase in contact time required in practices in SE deprived areas appears to be limited to male patients, but when the two sexes are combined, the adjustment required is similar to the overall calculation, at 2.1%. Broken down by age, different age bands are seen to require different adjustments, some positive and some negative, so that overall, the increase required in practices in SE deprived areas appears minimal, at 0.8%.

A different picture appears when stratifying the study population by number of LTCs. At every level of morbidity, patients in practices in SE deprived areas have less contact time with their practices, and practices would require a significant increase in resources to deliver the same level of care as practices in more SE affluent areas. The smallest increase required is 7.2%, for patients with the most complex needs, with five or more LTCs. When combined, these adjustments add up to an increase in contact time required in SE deprived areas of 14.4%.

Each of these calculations is stratified by only one condition, but the same calculations can use combinations of stratification factors, and these are shown in Table 3. Stratifying by age and sex alone, reveals that very little adjustment is required to achieve parity in terms of mean contact time. However, as soon as the number of LTCs are factored into the calculations, it is clear that a large increase in resources would be required for practices in SE deprived areas to offer the same amount of contact time to patients with the same level of need. Stratifying by age, sex, and number of LTCs, the estimated adjustment required is an increase of 13.5%.

For peer review only

Discussion

Summary

Using patient records from a sample of 150 Scottish general practices we demonstrate that substantially less care is received by patients in practices in socio-economically deprived areas compared to patients in affluent areas, but only when viewed in relation to morbidity, as measured by the number of LTCs. If general practices in highly SE deprived areas were to give an equivalent

For peer review only - http://bmjopen.bmj.com/site/about/guidelines.xhtml

amount of time (direct contact) to patients with the same level of need (the number of long-term conditions), they would require additional resources sufficient to support an increase of approximately 14% in patient contact time.

Strengths and limitations

This study utilises routine administrative data from a large population dataset which limits the potential bias associated with self-reported utilisation data[2] when seeking to measure unmet need. It demonstrates that data are retrievable from practice data systems to measure contact time with practice staff and allow for an estimation of resources required. The findings begin to address a universal problem about unmet need and resource allocation in primary care. However, our analysis excludes patients who made no appointments with their practice over the 3-year period. This may slightly bias our estimates of the average time spent with practice staff. However, such patients are likely to have very low health care need, and will have little impact on the resource requirements of their practices.

The accuracy of estimated time spent with patients has also not been ascertained in research, but we have reduced any inaccuracies by using record opening times to best reflect clinical contacts.[12] Outside the Quality and Outcomes Framework,[16] there is also some variation in how long-term health conditions are coded. However, we followed established methods to mitigate for potential errors.[6] We acknowledge that each long term condition included does not have equivalence in terms of impact on patients and that even within each condition there will be variation in the health care needs of individuals.[17]

The measure of socio-economic deprivation used is area-based, rather than one measured at an individual level to maximise accuracy; however this was the best available. Likewise we were unable – due to the anonymised nature of practice recruitment – to recruit a sample that was a perfect

For peer review only - http://bmjopen.bmj.com/site/about/guidelines.xhtml

representation of the population; however our TTP recruited practices based on our specific request to ensure an urban/rural and affluent/SE deprived area spread.

The lack of accurate information about ethnicity is a limitation. In our previous work,[11] we found that only 2.7% of appointments could be linked to ethnicity data in the medical records.

Provision of medical care by general practices does have some distinct features depending on practice locality. For example, in remote rural settings practices provide more pre-hospital emergency care which means more time may be spent with patients in those circumstances and this may bias consultation time estimates in these areas.

Finally these data predate the rapid and far reaching changes to general practice access in the UK triggered by the Covid pandemic. It will be some time yet before the longer-term consequences and eventual access arrangements settle into a new normal and their consequences are understood.

Comparison with existing literature

Research from Scotland over 20 years ago, and more recently, observed that consultations in more socio-economically deprived areas tended to be shorter[18, 19] and these findings have been replicated in England.[20] We know that provision of GP resource is flatly distributed in Scotland[21] and there are indications that the revised Scottish GP contract funding formula from 2018 has not had a positive impact in more SE deprived areas.[22] An in-depth analysis conducted with English routine administrative data showed that practices in high SE deprivation areas have fewer GPs and more practice nurses than affluent area-based counterparts. This means GPs in those settings are caring for 10% more patients overall.[22] What this paper demonstrates, for the first time, is how clinically focussed evidence that relies on high quality data from a large percentage of the Scottish population can inform and improve models of care. Put simply, a patient registered at a practice serving an affluent population will receive more care from their practice than a patient of the same age, sex, and level of morbidity, registered with a practice serving a more SE deprived population.

For peer review only

Implications for research and/or practice

This study adds to growing evidence that provision of general practice care in the UK is not equitably distributed across practices and needs to change.[24] Patients with long-term health conditions living in the least affluent areas are losing out. This is unacceptable unmet need because it is systematically related to socio-economic factors.

We estimate a 14% increase in clinical staff contact time is required in SE deprived areas to equalise provision. A direct impact of this analysis is that governments, health service policy makers and planners should consider using data on contact time to measure the amount of care delivered to patients, as well as data on the number of recorded LTCs to estimate the level of need of GP practice populations. This is now achievable through routine administrative data capture and should be factored into resource allocation algorithms. This paper adds to the large body of work now accumulated in the UK about the role general practice care has in perpetuating health inequalities and the inverse care law.[25]

Conclusion

General practice provision in SE deprived areas needs to be better supported to deliver uniform care to patients with the same level of need across the UK. The analysis from this paper suggests that measuring LTCs and funding accordingly would make a significant contribution to achieving this aim.

Ethics approval

Letters of comfort were issued by the West of Scotland NHS Ethics Committee and the University of Glasgow College of Medical, Veterinary & Life Sciences Ethics Committee confirming that the full study did not need NHS ethics approval as this used routine data that had been extracted in an anonymised format. Public Benefit and Privacy Panel approval for use of the data was granted by NHS Information Services Scotland in December 2016.

For peer review only

For peer review only - http://bmjopen.bmj.com/site/about/guidelines.xhtml

Author contributions

AEW: Designed and managed the study, wrote first draft of manuscript, reviewed final version of manuscript. DE, PW: Designed and managed the study, reviewed final version of manuscript. RM: Processed data for analysis, reviewed final version of manuscript. AM: Conceived and carried out statistical analysis, wrote statistical methods and results, reviewed final version of manuscript.

Data Availability

These data were available from NHS Scotland and access permissions were contingent on them not being publicly available. New data access and TTP requests can be made to www.escro.co.uk for general practice data and https://www.isdscotland.org/products-and-services/edris/ to host the data securely and request data linkage. The authors had no special access permissions that others could not have. Analysis code is available from the authors upon reasonable request. The code will replicate the study findings, but the analysis can be replicated using other statistical software.

Acknowledgements

Thank you to all the GP practices who participated in the Serial Missed Appointments study and for strategic support from Ellen Lynch (Health and Social Care Analytical Services, Scottish Government). The general practice data expertise of Dave Kelly (Albasoft) was invaluable. Thanks also to the eDRIS team who facilitated the safe use of our data in the Safehaven, especially Dionysis Vragkos.

Study Protocol

See protocol paper.[12]

Funding

This work was supported by a grant from Chief Scientist Office, Scottish Government (reference CZH/4/1118) with Safe Haven and data linkage costs supported in lieu by the DSLS at Scottish Government. The funding sources for this analysis had no influence over study design, data

For peer review only

collection, data analysis, data interpretation, the writing of the report, or the decision to submit for publication.

Competing Interests

None declared

Figure Legends

Figure 1: Mean duration of appointments (over 3 years), by age, sex, and number of long-term conditions, for practices with SE deprived and affluent populations.

For peer review only

For peer review only - http://bmjopen.bmj.com/site/about/guidelines.xhtml

References

1. Smith S, Connolly S. Re-thinking unmet need for health care: introducing a dynamic perspective. *Health Econ Policy Law*. 2020;15(4):440-57.
2. Allin S, Grignon M, Le Grand J. Subjective unmet need and utilization of health care services in Canada: What are the equity implications? *Soc Sci Med*. 2010;70(3):465-72.
3. Pearson-Stuttard J, Ezzati M, Gregg EW. Multimorbidity-a defining challenge for health systems. *Lancet Public Health*. 2019;4(12):e599-e600.
4. Whitty CJM, MacEwen C, Goddard A, et al. Rising to the challenge of multimorbidity. *BMJ*. 2020;368:l6964.
5. Rolewicz L, Keeble E, Paddison C, et al. Are the needs of people with multiple long-term conditions being met? Evidence from the 2018 General Practice Patient Survey. *BMJ Open*. 2020;10(11):e041569.
6. Barnett K, Mercer SMN, Watt G, et al. Epidemiology of Multi-morbidity and implications for health care, research and medical education: a cross sectional study. *Lancet*. 2012;380((9836)):37-43.
7. Menotti A, Mulder I, Nissinen A, et al. Prevalence of morbidity and multimorbidity in elderly male populations and their impact on 10-year all-cause mortality: The FINE study (Finland, Italy, Netherlands, Elderly). *J Clin Epidemiol*. 2001;54(7):680-6.
8. Stanley J, Semper K, Millar E, et al. Epidemiology of multimorbidity in New Zealand: a cross-sectional study using national-level hospital and pharmaceutical data. *BMJ Open*. 2018;8(5):e021689.
9. Sullivan MK, Jani BD, McConnachie A, et al. Hospitalisation events in people with chronic kidney disease as a component of multimorbidity: parallel cohort studies in research and routine care settings. *BMC Med.* 2021;19(1):278.
10. Albasoft, role and responsibilities as an NHS Trusted Third Party [Available from: http://www.escro.co.uk/marketing/TTP%20in%20primary%20care%20summary.pdf.
11. Ellis DA, McQueenie R, McConnachie A, et al. Demographic and practice factors predicting repeated non-attendance in primary care: a national retrospective cohort analysis. *Lancet Public Health*. 2017;2.
12. Williamson AE, Ellis DA, Wilson P, et al. Understanding repeated non-attendance in health services: a pilot analysis of administrative data and full study protocol for a national retrospective cohort. *BMJ Open*. 2017;7.
13. McQueenie R, Ellis DA, McConnachie A, et al. Morbidity, mortality and missed appointments in healthcare: a national retrospective data linkage study. *BMC Med*. 2019;17(1):2.
14. Scottish Index of Multiple Deprivation 2012 [Internet]. Scottish Government. 2012. Available from: http://www.gov.scot/Topics/Statistics/SIMD/DataAnalysis/Background-Data-2012.
15. R Core Team (2021). R: A language and environment for statistical computing. R Foundation for Statistical Computing, Vienna, Austria. URL https://www.R-project.org/.
16. Information Services Division NHS Scotland. General Practice Quality and Outcomes Framework UK: Public Health Scotland; 2020 [Available from: https://www.isdscotland.org/health-topics/general-practice/quality-and-outcomes-framework/.
17. Johnston MC, Crilly M, Black C, et al. Defining and measuring multimorbidity: a systematic review of systematic reviews. *Eur J Public Health*. 2019;29(1):182-9.
18. Stirling AM, Wilson P, McConnachie A. Deprivation, psychological distress, and consultation length in general practice. *Br J Gen Pract*. 2001;51(467):456-60.
19. Mercer SW, Watt GCM. The inverse care law: clinical primary care encounters in deprived and affluent areas of Scotland. *Ann Fam Med*. 2007;5(6):503-10.
20. Gopfert A, Deeny SR, Fisher R, et al. Primary care consultation length by deprivation and multimorbidity in England: an observational study using electronic patient records. *Br J Gen Pract*. 2021;71(704):e185-e92.

For peer review only

For peer review only - http://bmjopen.bmj.com/site/about/guidelines.xhtml

21. McLean G, Guthrie B, Mercer SW, et al. General practice funding underpins the persistence of the inverse care law: cross-sectional study in Scotland. *Br J Gen Pract*. 2015;65(641):e799-e805.
22. Morton C. Health Inequalities in Scotland A National Calamity; a Frontline GP view. UK: Independently published; 2020.
23. Fisher R, Dunn P, Asaria M, et al. Level or not? Comparing general practice in areas of high and low socioeconomic deprivation in England UK; 2020.
24. Ashworth M, L'Esperance V, Round T. Primary care funding entrenches health inequalities: time for a rethink. *Br J Gen Pract*. 2021;71(704):102-4.
25. Cookson R, Doran T, Asaria M, et al. The inverse care law re-examined: a global perspective. Lancet. 2021;397(10276):828-38.

For peer review only

For peer review only - http://bmjopen.bmj.com/site/about/guidelines.xhtml

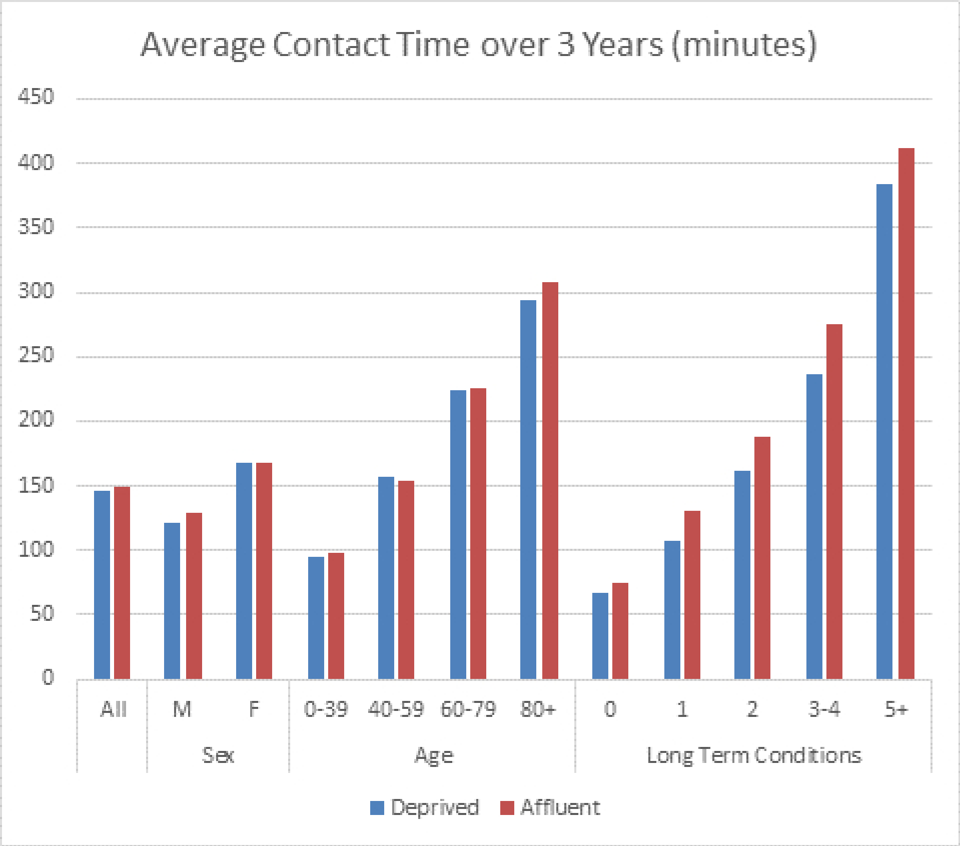

Mean duration of appointments (over 3 years), by age, sex, and number of long-term conditions, for practices with SE deprived and affluent populations.

127x111mm (96 x 96 DPI)

The RECORD statement – checklist of items, extended from the STROBE statement, that should be reported in observational studies using routinely collected health data.

	Item No.	STROBE items	Location in manuscript where items are reported	RECORD items	Location in manuscript where items are reported
**Title and abstract**					
	1	(a) Indicate the study's design with a commonly used term in the title or the abstract<br><br>(b) Provide in the abstract an informative and balanced summary of what was done and what was found	Title<br><br>p2, line 20-42	RECORD 1.1: The type of data used should be specified in the title or abstract. When possible, the name of the databases used should be included.<br><br>RECORD 1.2: If applicable, the geographic region and timeframe within which the study took place should be reported in the title or abstract.<br><br>RECORD 1.3: If linkage between databases was conducted for the study, this should be clearly stated in the title or abstract.	Title<br><br>p2, line 26-28<br><br>N/A
**Introduction**					
Background rationale	2	Explain the scientific background and rationale for the investigation being reported			p4, line 60-82
Objectives	3	State specific objectives, including any prespecified hypotheses			p4, line 80-82
**Methods**					
Study Design	4	Present key elements of study design early in the paper			Title<br>p5, line 84-96
Setting	5	Describe the setting, locations, and relevant dates, including periods of recruitment, exposure, follow-up, and data collection			p5, line 84-96

For peer review only

Participants	6	*(a) Cohort study* - Give the eligibility criteria, and the sources and methods of selection of participants. Describe methods of follow-up<br>*Case-control study* - Give the eligibility criteria, and the sources and methods of case ascertainment and control selection. Give the rationale for the choice of cases and controls<br>*Cross-sectional study* - Give the eligibility criteria, and the sources and methods of selection of participants	p5, line 84-103	RECORD 6.1: The methods of study population selection (such as codes or algorithms used to identify subjects) should be listed in detail. If this is not possible, an explanation should be provided.<br><br>RECORD 6.2: Any validation studies of the codes or algorithms used to select the population should be referenced. If validation was conducted for this study and not published elsewhere, detailed methods and results should be provided.	p5, line 91-95<br><br>N/A
		(b) Cohort study - For matched studies, give matching criteria and number of exposed and unexposed<br>*Case-control study* - For matched studies, give matching criteria and the number of controls per case	N/A	RECORD 6.3: If the study involved linkage of databases, consider use of a flow diagram or other graphical display to demonstrate the data linkage process, including the number of individuals with linked data at each stage.	N/A
Variables	7	Clearly define all outcomes, exposures, predictors, potential confounders, and effect modifiers. Give diagnostic criteria, if applicable.	p5-6, line 97-113	RECORD 7.1: A complete list of codes and algorithms used to classify exposures, outcomes, confounders, and effect modifiers should be provided. If these cannot be reported, an explanation should be provided.	p16, line 274-275
Data sources/ measurement	8	For each variable of interest, give sources of data and details of methods of assessment (measurement).<br>Describe comparability of assessment methods if there is more than one group			p5-6, line 84-113

For peer review only - http://bmjopen.bmj.com/site/about/guidelines.xhtml

Bias	9	Describe any efforts to address potential sources of bias			p13-14, line 197-224
Study size	10	Explain how the study size was arrived at			N/A
Quantitative variables	11	Explain how quantitative variables were handled in the analyses. If applicable, describe which groupings were chosen, and why			p5, line 98-103
Statistical methods	12	(a) Describe all statistical methods, including those used to control for confounding<br>(b) Describe any methods used to examine subgroups and interactions<br>(c) Explain how missing data were addressed<br>(d) *Cohort study* - If applicable, explain how loss to follow-up was addressed<br>*Case-control study* - If applicable, explain how matching of cases and controls was addressed<br>*Cross-sectional study* - If applicable, describe analytical methods taking account of sampling strategy<br>(e) Describe any sensitivity analyses			p6, line 114-119
Data access and cleaning methods		..		RECORD 12.1: Authors should describe the extent to which the investigators had access to the database population used to create the study population.	p5, line 97

For peer review only

				RECORD 12.2: Authors should provide information on the data cleaning methods used in the study.	p5, line 97
Linkage		..		RECORD 12.3: State whether the study included person-level, institutional-level, or other data linkage across two or more databases. The methods of linkage and methods of linkage quality evaluation should be provided.	N/A
**Results**					
Participants	13	(a) Report the numbers of individuals at each stage of the study (*e.g.*, numbers potentially eligible, examined for eligibility, confirmed eligible, included in the study, completing follow-up, and analysed)<br>(b) Give reasons for non-participation at each stage.<br>(c) Consider use of a flow diagram	p6-7, line 128-133	RECORD 13.1: Describe in detail the selection of the persons included in the study (*i.e.,* study population selection) including filtering based on data quality, data availability and linkage. The selection of included persons can be described in the text and/or by means of the study flow diagram.	p6-7, line 128-133
Descriptive data	14	(a) Give characteristics of study participants (*e.g.*, demographic, clinical, social) and information on exposures and potential confounders<br>(b) Indicate the number of participants with missing data for each variable of interest<br>(c) *Cohort study* - summarise follow-up time (*e.g.*, average and total amount)			p7, line 133-139
Outcome data	15	*Cohort study* - Report numbers of outcome events or summary measures over time<br>*Case-control study* - Report numbers in each exposure			p10, line 146-168

For peer review only

		category, or summary measures of exposure<br>*Cross-sectional study* - Report numbers of outcome events or summary measures			
Main results	16	(a) Give unadjusted estimates and, if applicable, confounder-adjusted estimates and their precision (e.g., 95% confidence interval). Make clear which confounders were adjusted for and why they were included<br>(b) Report category boundaries when continuous variables were categorized<br>(c) If relevant, consider translating estimates of relative risk into absolute risk for a meaningful time period			p10-12, line 162-187
Other analyses	17	Report other analyses done—e.g., analyses of subgroups and interactions, and sensitivity analyses			p10-12, line 162-187
**Discussion**					
Key results	18	Summarise key results with reference to study objectives			p12-13, line 189-196
Limitations	19	Discuss limitations of the study, taking into account sources of potential bias or imprecision. Discuss both direction and magnitude of any potential bias	p13-14, line 197-227	RECORD 19.1: Discuss the implications of using data that were not created or collected to answer the specific research question(s). Include discussion of misclassification bias, unmeasured confounding, missing data, and changing eligibility over time, as they pertain to the study being reported.	p13-14, line 197-227
Interpretation	20	Give a cautious overall interpretation of results			p14, line 228-240

For peer review only

		considering objectives, limitations, multiplicity of analyses, results from similar studies, and other relevant evidence			
Generalisability	21	Discuss the generalisability (external validity) of the study results			p13-14, line 214-224
**Other Information**					
Funding	22	Give the source of funding and the role of the funders for the present study and, if applicable, for the original study on which the present article is based			p16-17, line 283-288
Accessibility of protocol, raw data, and programming code		..		RECORD 22.1: Authors should provide information on how to access any supplemental information such as the study protocol, raw data, or programming code.	p16, line 281-282<br>p16, line 269-275

*Reference: Benchimol EI, Smeeth L, Guttmann A, Harron K, Moher D, Petersen I, Sørensen HT, von Elm E, Langan SM, the RECORD Working Committee. The REporting of studies Conducted using Observational Routinely-collected health Data (RECORD) Statement. *PLoS Medicine* 2015; in press.

*Checklist is protected under Creative Commons Attribution (CC BY) license.

For peer review only

BMJ Open

Quantifying unmet need in General Practice: a retrospective cohort study of administrative data

Journal:	*BMJ Open*
Manuscript ID	bmjopen-2022-068720.R2
Article Type:	Original research
Date Submitted by the Author:	27-Apr-2023
Complete List of Authors:	McConnachie, Alex; University of Glasgow, Robertson Centre for Biostatistics<br>Ellis, David; University of Bath, School of Management<br>Wilson, Philip; University of Aberdeen, Institute of Health and Wellbeing<br>McQueenie, Ross; University of Glasgow, Genera Practice and Primary Care<br>Williamson, Andrea; University of Glasgow, GPPC, School of Medicine, Dentistry and Nursing, MVLS
**Primary Subject Heading**:	General practice / Family practice
Secondary Subject Heading:	Health services research
Keywords:	PRIMARY CARE, Organisation of health services < HEALTH SERVICES ADMINISTRATION & MANAGEMENT, Quality in health care < HEALTH SERVICES ADMINISTRATION & MANAGEMENT

SCHOLARONE™
Manuscripts

BMJ

I, the Submitting Author has the right to grant and does grant on behalf of all authors of the Work (as defined in the below author licence), an exclusive licence and/or a non-exclusive licence for contributions from authors who are: i) UK Crown employees; ii) where BMJ has agreed a CC-BY licence shall apply, and/or iii) in accordance with the terms applicable for US Federal Government officers or employees acting as part of their official duties; on a worldwide, perpetual, irrevocable, royalty-free basis to BMJ Publishing Group Ltd ("BMJ") its licensees and where the relevant Journal is co-owned by BMJ to the co-owners of the Journal, to publish the Work in this journal and any other BMJ products and to exploit all rights, as set out in our licence.

The Submitting Author accepts and understands that any supply made under these terms is made by BMJ to the Submitting Author unless you are acting as an employee on behalf of your employer or a postgraduate student of an affiliated institution which is paying any applicable article publishing charge ("APC") for Open Access articles. Where the Submitting Author wishes to make the Work available on an Open Access basis (and intends to pay the relevant APC), the terms of reuse of such Open Access shall be governed by a Creative Commons licence – details of these licences and which Creative Commons licence will apply to this Work are set out in our licence referred to above.

Other than as permitted in any relevant BMJ Author's Self Archiving Policies, I confirm this Work has not been accepted for publication elsewhere, is not being considered for publication elsewhere and does not duplicate material already published. I confirm all authors consent to publication of this Work and authorise the granting of this licence.

For peer review only

Quantifying unmet need in General Practice: a retrospective cohort study of administrative data

Alex McConnachie[1], David A Ellis[2], Philip Wilson[3], Ross McQueenie[4], Andrea E Williamson[5]

[1]BSc MSc PhD, Professor of Clinical Trial Biostatistics, Robertson Centre for Biostatistics, School of Health and Wellbeing, MVLS, University of Glasgow, Scotland, UK. ORCID ID: 0000-0002-7262-7000

[2] MA MSc PhD, Associate Professor in Information Systems; School of Management, University of Bath, Claverton Down, Bath BA2 7AY, UK. ORCID ID: 0000-0001-6172-3323

[3] MA MB BChir DPhil DCH FRCPCH FRCGP, Professor emeritus, Institute of Applied Health Sciences, University of Aberdeen, Scotland, UK. ORCID ID: 0000-0002-4123-8248

[4] BSc Hons, PhD, Research Associate, General Practice and Primary Care, School of Health and Wellbeing, MVLS, University of Glasgow, 1 Horselethill Road, Glasgow, G12 9LX UK. ORCID ID: 0000-0002-0070-6607

[5] MBChB MPH PhD FRCGP FHEA DTM&H, Professor of General Practice and Inclusion Health, General Practice and Primary Care, School of Health and Wellbeing, MVLS, University of Glasgow, 1 Horselethill Road, Glasgow, G12 9LX UK. ORCID ID: 0000-0002-8981-9068 @aewilliamsonl

Correspondence to: Professor Alex McConnachie, Robertson Centre for Biostatistics, School of Health and Wellbeing, MVLS, University of Glasgow, Scotland, UK, alex.mcconnachie@glasgow.ac.uk

Word count (excluding abstract, references, tables, figures): 3277

For peer review only

For peer review only - http://bmjopen.bmj.com/site/about/guidelines.xhtml

Abstract

Objectives: To assess whether patients attending General Practices (GPs) in socioeconomically deprived areas receive the same amount of care, compared to similar patients (based on age, sex, and level of morbidity) attending GPs in less deprived areas. If not, to quantify the additional resource that would be required by GPs in deprived areas to achieve parity.

Design: Retrospective cohort study.

Setting: 150 General Practices in Scotland, UK, divided into two groups: 80 practices in Scottish Index of Multiple Deprivation (SIMD) decile 1-5 (more SE deprived); 70 practices in SIMD decile 6-10 (less SE deprived).

Patients: 437,590 patients registered with a more SE deprived GP, and 333,994 patients registered with a less SE deprived GP, for the whole study period (2013-2016), who made at least one appointment.

Outcomes: The number of contacts and total contact time between patients and clinical staff.

Results: Patients in more SE deprived areas had slightly more discrete contacts over 3 years (11.8 vs. 11.4), but each patient had marginally less contact time (146.1 vs 149.5 minutes). Stratified by sex and age, differences were also small. Stratified by the number of Long-Term Conditions (nLTCs), practices in more SE deprived areas delivered significantly less contact time than practices in less SE deprived areas. Over three years, 8 fewer minutes for patients with no LTCs, and 24, 27, 38, and 28 fewer minutes for patients with 1, 2, 3-4, or 5+ LTCs, respectively.

Conclusion: If GPs in more SE deprived areas were to give an equal amount of direct contact time to patients with the same level of need served by GPs in less SE deprived areas, this would require a 14% increase in patient contact time. This represents a significant unmet need, supporting the case for redistribution of resources to tackle the inverse care law.

Keywords: general practice, health care planning, unmet need, inverse care law, inequalities, big data

For peer review only

For peer review only - http://bmjopen.bmj.com/site/about/guidelines.xhtml

Strengths and limitations

Strengths

- Large dataset, based on routine administrative records, demonstrating the feasibility of measuring contact time with practice staff.
- Large sample of practices covering urban and rural, affluent and deprived areas.

Limitations

- The accuracy of time spent with patients based on the opening and closing of medical records has not been verified.
- Health care need is estimated by a simple count of the number of long-term conditions, which does not reflect variation in health care needs between conditions, nor variation in needs between patients within the same condition.
- These data predate the rapid and far reaching changes to general practice access in the UK triggered by the Covid pandemic.

For peer review only

Introduction

Unmet need is a multidimensional, complex construct;[1] "*when an individual does not receive an available and effective treatment that could have improved her health…some unmet need is acceptable since resources are scarce. What is of concern here is whether unmet need is inequitable, or systematically related to socioeconomic or other personal characteristics*".[2]

Patients living with multi-morbidity (two or more co-existing long-term health conditions (LTCs)) have complex needs, and successfully meeting those within our healthcare systems is one of the most pressing challenges of our time.[3-5] We already have strong evidence that people living in more socio-economically (SE) deprived areas in the UK have a burden of multi-morbidity at an earlier age.[6] Furthermore, patients with multi-morbidity are at greater risk of all-cause mortality[7, 8] and hospitalisation,[8] regardless of whether the number of LTCs are measured in a research setting or via primary care records,[9] suggesting that the numbers of LTCs recorded in general practice electronic health records broadly reflect underlying healthcare need.

The analyses presented in this paper stem from research exploring the determinants and outcomes of missed appointments in general practice in Scotland. During this work, we determined the total number and duration of contacts between clinical practice staff and individual patients. We postulated that patients with more LTCs have greater healthcare need, and therefore require more contact time with clinical practice staff. In an equitable health service, equivalent patients (according to their age, sex, and number of LTCs) should, on average, receive the same amount of contact with clinical practice staff, regardless of whether they live in an affluent or socio-economically (SE) deprived area. Therefore, we hypothesised that patients in SE deprived communities may receive less clinical GP staff contact time with an equivalent level of morbidity. If this is the case, how much more contact time would be required in socio-economically deprived practices to achieve parity with affluent practices?

For peer review only - http://bmjopen.bmj.com/site/about/guidelines.xhtml

Methods

A Trusted Third party (TTP) for the National Health Service (NHS) in Scotland (Albasoft), recruited general practices (GPs) on our behalf. A TTP is an organisation independent from the NHS with the skills, infrastructure and permissions to extract and link data safely without having a direct interest in the use to which the data will be put. The TTP is required to ensure confidentiality for patients and professionals in the research process.[10] The TTP recruited practices to ensure a spread of practices in urban and rural locations, serving both affluent and socio-economically (SE) deprived (Deep End) populations. All patients who were alive and registered at the same practice for the 3-year period (5th September 2013 to 5th September 2016) and who made at least one appointment with the practice were included in the data set. All patients retained also had data on age, sex, number of recorded LTCs, and the number and total duration of all in person contacts with clinical practice staff such as GPs and practice nurses. Recording of patient ethnicity and language interpreter requirements was very low so this could not be included in our analysis.[11]

How we defined and measured LTCs are documented in previous publications.[11-13]

We present the number and percentage of patients in practices serving affluent and deprived populations, according to sex, age (0-39, 40-59, 60-79, and 80+ years), and the number of LTCs (none, 1, 2, 3-4, or 5+). Practices were categorised as affluent or deprived based on the average Scottish Index of Multiple Deprivation (SIMD) score of all patients registered at the practice. SIMD is routinely used in Scotland as part of the current GP contract funding allocation formula. When split by decile groups, 1 is the most SE deprived.[14]

We calculated the total number and duration of contacts in minutes for each patient. These were extracted from appointments recorded via practice IT systems. The duration of contacts was calculated from the 'in' and 'out' time. Detailed proof of concept work in our pilot study of 67,705 patient records enabled us to set rules about included appointments to increase accuracy.

For peer review only - http://bmjopen.bmj.com/site/about/guidelines.xhtml

Appointments that were 'open' for less than 2 minutes, 'open' for more than 24 hours, labelled as an administrative slot, or were not face-to-face appointments were excluded.[12] There is no unified categorisation of General Practice appointment types in the UK and this was reflected in the heterogeneity of appointment categories in our data set. Therefore, all appointments included were for face-to-face appointments with a health care professional in the practice (rather than being specific e.g. about GP or practice nurse contacts).[11]

We present the mean and standard deviation (SD) of the number and duration of contacts for patients in practices in affluent and deprived areas, according to sex, age, and the number of LTCs.

Taking the mean contact time provided by practices in affluent areas and applying this to the number of patients served by practices in SE deprived areas, we calculated the change in total contact time that would be required in practices in SE deprived areas in order to give the same average contact time. We then repeated this analysis, stratified by sex, age, and/or number of LTCs.

All analyses were done using the R statistical software package [15].

Patient and Public Involvement

The Royal College of General Practitioners Scotland Patient Partnership in Practice (P^3) Committee in 2016 provided advice about the relevance and importance of this research. This was to inform the study team's application for data linkage of routine data sets. The P^3 committee (a lay patient group) advised that they agreed it was 'interesting, much needed and beneficial' and they have been updated on progress as the project has proceeded.

For peer review only

Results

Data from 150 Scottish practices were included. These were split into two groups: SIMD decile 1-5 (most SE deprived, 80 practices) and SIMD decile 6-10 (least deprived, 70 practices). From a cohort of 824,374 patients, after exclusion of patients who moved practice, or who had missing data for age,

For peer review only - http://bmjopen.bmj.com/site/about/guidelines.xhtml

sex, or number of LTCs, data from 771,584 patient records were analysed. The study population was 437,590 patients from the 80 practices in SE deprived areas, and 333,994 patients from the 70 practices in affluent areas. **Table 1** sets out their characteristics. Differences in terms of the sex and age distributions are small. 47% of patients from SE deprived practices in this sample were male, compared to 46% of patients in practices in more affluent areas. Patients registered with practices in SE deprived areas were slightly younger, with 48% vs. 47% being under 40, and 4.6% vs. 5.2% being over 80 years of age. The difference in terms of the distribution of the number of LTCs that patients had was far more marked, with 45% of patients in practices serving affluent populations having no LTCs, compared to 37% of patients in practices serving SE deprived populations.

Table 1: Study population. Numbers and percentages of patients in groups defined by sex, age, and number of long-term conditions, for practices with SE deprived and affluent populations.

		Practices in Deprived Areas	Practices in Affluent Areas
Number of Practices		80	70
Number of Patients		437,590	333,994
Sex			
Male	N (%)	205,914 (47.1%)	155,289 (46.5%)
Female	N (%)	231,676 (52.9%)	178,705 (53.5%)
Age Group			
0-39 years	N (%)	211,917 (48.4%)	157,731 (47.2%)
40-59 years	N (%)	123,385 (28.2%)	93,368 (28.0%)
60-79 years	N (%)	82,315 (18.8%)	65,559 (19.6%)
80+ years	N (%)	19,973 (4.6%)	17,336 (5.2%)
Number of Long-Term Conditions			
0	N (%)	163,616 (37.4%)	151,063 (45.2%)
1	N (%)	98,470 (22.5%)	74,497 (22.3%)
2	N (%)	63,847 (14.6%)	43,597 (13.1%)
3-4	N (%)	72,814 (16.6%)	44,006 (13.2%)
5+	N (%)	38,843 (8.9%)	20,831 (6.2%)

Table 2 shows the mean number and duration of contacts between patients and clinical practice staff, by age, sex, and number of LTCs, and the estimated differences between practices serving a

For peer review only

For peer review only - http://bmjopen.bmj.com/site/about/guidelines.xhtml

population living in relatively SE deprived areas, and practices serving more affluent areas. Confidence intervals are derived from simple two-sample t-tests. **Figure 1** displays mean contact time by age, sex, and number of LTCs, for practices with SE deprived and affluent populations.

For peer review only

Table 2: Mean number and duration of appointments (over 3 years), by age, sex, and number of long-term conditions, for practices with SE deprived and affluent populations.

	Total Number of Appointments			Total Duration of Appointments (Minutes)		
	Practices in Deprived Areas	Practices in Affluent Areas	Difference (95% CI), p-value	Practices in Deprived Areas	Practices in Affluent Areas	Difference (95% CI), p-value
	Mean (SD)	Mean (SD)		Mean (SD)	Mean (SD)	
All	11.8 (14.6)	11.4 (14.2)	0.4 (0.3, 0.5), p<0.0001	146.1 (228.4)	149.5 (223.4)	-3.3 (-4.4, -2.3), p<0.0001
Sex						
Male	9.9 (13.2)	9.9 (13.3)	0.0 (-0.1, 0.1), p=0.4361	121.1 (207.0)	128.5 (209.6)	-7.4 (-8.8, -6.0), p<0.0001
Female	13.5 (15.6)	12.7 (14.8)	0.8 (0.7, 0.9), p<0.0001	168.4 (243.7)	167.7 (233.2)	0.7 (-0.8, 2.2), p=0.36
Age						
0-39 years	8.2 (9.6)	7.6 (8.9)	0.5 (0.5, 0.6), p<0.0001	95.2 (135.1)	97.6 (143.4)	-2.4 (-3.3, -1.5), p<0.0001
40-59 years	12.7 (14.7)	11.3 (13.3)	1.3 (1.2, 1.5), p<0.0001	157.6 (222.6)	153.9 (223.4)	3.8 (1.9, 5.7), p=0.0001
60-79 years	17.6 (18.6)	17.3 (18.1)	0.3 (0.1, 0.5), p=0.0021	223.9 (299.5)	225.9 (284.8)	-2.0 (-5.0, 0.9), p=0.18
80+ years	21.8 (24.1)	24.3 (23.5)	-2.5 (-3.0, -2.0), p<0.0001	294.5 (445.1)	308.1 (356.6)	-13.6 (-21.7, -5.4), p=0.0011
Number of Long-Term Conditions						
0	5.9 (6.7)	6.0 (6.5)	-0.1 (-0.2, -0.1), p<0.0001	66.9 (95.6)	74.7 (101.5)	-7.9 (-8.6, -7.2), p<0.0001
1	9.2 (9.4)	10.1 (9.9)	-0.8 (-0.9, -0.7), p<0.0001	107.0 (134.7)	130.8 (157.7)	-23.8 (-25.3, -22.4), p<0.0001
2	13.4 (13.0)	14.1 (13.1)	-0.7 (-0.8, -0.5), p<0.0001	161.1 (195.9)	187.9 (223.1)	-26.8 (-29.4, -24.2), p<0.0001
3-4	18.8 (17.5)	20.5 (18.4)	-1.7 (-1.9, -1.5), p<0.0001	236.8 (267.6)	275.0 (305.2)	-38.2 (-41.7, -34.8), p<0.0001
5+	27.7 (25.2)	30.7 (26.9)	-3.0 (-3.5, -2.6), p<0.0001	384.6 (450.3)	412.3 (434.1)	-27.7 (-35.1, -20.3), p<0.0001

For peer review only

For peer review only - http://bmjopen.bmj.com/site/about/guidelines.xhtml

Overall, patients in SE deprived areas had more discrete contacts with clinical practice staff over 3 years (11.8 vs.11.4), but each patient had less contact time (146.1 vs 149.5 minutes). Men in SE deprived areas had the same number of contacts, but spent 7.4 minutes less time with clinical staff, compared to men in affluent areas. Women in practices in SE deprived areas had slightly more contacts than women in affluent area practices (0.8 more contacts over 3 years); but spent a similar amount of time with clinical practice staff.

Patterns by age are more complex. For the youngest age group (under 40 years), and for those aged 60-70 years, patients in SE deprived area practices had marginally more contacts, but less time with clinical practice staff. In the 40-59 age band, however, patients in SE deprived areas had slightly more contacts, and spent slightly more time with clinical practice staff. In the oldest patients (over 80 years) patients registered to practices serving more SE deprived populations had fewer contacts and spent less time with the practice.

However, when looking at the data in relation to the number of LTCs that were recorded, there is a consistent and striking pattern. Patients in practices in SE deprived areas clearly have fewer contacts, and spend less time with clinical practice staff, compared to patients in more affluent practices, at every level of morbidity.

Table 3 shows how much contact time would be required between patients and staff in practices serving more SE deprived populations if the average contact time were the same as in practices with more affluent populations. Overall, practices in affluent areas spent about 50 million minutes with 334,000 patients, at an average of roughly 150 minutes per patient over the three-year study period. This was slightly more than the average contact time in practices in SE deprived areas, so that if these practices were to have the same average contact time, the total amount of time spent in contact with patients would need to increase by 2.3%.

For peer review only

For peer review only - http://bmjopen.bmj.com/site/about/guidelines.xhtml

Table 3: Additional resource requirements (in terms of total appointment time) for General Practices in deprived areas to offer equal appointment times to General Practices in affluent areas

	Practices in Affluent Areas (N=70)		Practices in Deprived Areas (N=80)			
	Observed Appointment Times		Observed Appointment Times		Assuming Equitable Appointment Times	
**Stratification**	Total Minutes	Mean	Total Minutes	Mean	Total minutes	% Change Required
**None**	49,919,411	149.5	63,942,072	146.1	65,403,076	+2.3%
**Sex** – Male	19,951,031	128.5	24,930,987	121.1	26,455,168	+6.1%
**Sex** – Female	29,968,380	167.7	39,011,085	168.4	38,851,484	-0.4%
**Sex** – Overall					65,306,652	+2.1%
**Age** - 0-39	15,401,793	97.6	20,180,456	95.2	20,692,836	+2.5%
**Age** - 40-59	14,365,312	153.9	19,450,870	157.6	18,983,635	-2.4%
**Age** - 60-79	14,811,401	225.9	18,428,732	223.9	18,596,996	+0.9%
**Age** - 80+	5,340,905	308.1	5,882,014	294.5	6,153,317	+4.6%
**Age** - Overall					64,426,784	+0.8%
**LTC Count** – 0	11,291,456	74.7	10,942,172	66.9	12,229,751	+11.8%
**LTC Count** – 1	9,744,067	130.8	10,531,390	107.0	12,879,690	+22.3%
**LTC Count** – 2	8,192,478	187.9	10,285,517	161.1	11,997,732	+16.6%
**LTC Count** – 3-4	12,103,247	275.0	17,244,417	236.8	20,026,492	+16.1%
**LTC Count** – 5+	8,588,163	412.3	14,938,576	384.6	16,014,114	+7.2%
**LTC Count** – Overall					73,147,780	+14.4%
**Age, Sex** – Overall					64,311,097	+0.6%
**Sex, LTC Count** – Overall					72,988,341	+14.1%
**Age, LTC Count** – Overall					72,834,600	+13.9%
**Age, Sex, LTC Count** - Overall					72,563,329	+13.5%

For peer review only

For peer review only - http://bmjopen.bmj.com/site/about/guidelines.xhtml

The same calculations can be stratified by sex. The increase in contact time required in practices in SE deprived areas appears to be limited to male patients, but when the two sexes are combined, the adjustment required is similar to the overall calculation, at 2.1%. Broken down by age, different age bands are seen to require different adjustments, some positive and some negative, so that overall, the increase required in practices in SE deprived areas appears minimal, at 0.8%.

A different picture appears when stratifying the study population by number of LTCs. At every level of morbidity, patients in practices in SE deprived areas have less contact time with their practices, and practices would require a significant increase in resources to deliver the same amount of care as practices in more SE affluent areas. The smallest increase required is 7.2%, for patients with the most complex needs, with five or more LTCs. When combined, these adjustments add up to an increase in contact time required in SE deprived areas of 14.4%.

Each of these calculations is stratified by only one condition, but the same calculations can use combinations of stratification factors, and these are shown in Table 3. Stratifying by age and sex alone, reveals that very little adjustment is required to achieve parity in terms of mean contact time. However, as soon as the number of LTCs are factored into the calculations, it is clear that a large increase in resources would be required for practices in SE deprived areas to offer the same amount of contact time to patients with the same level of need. Stratifying by age, sex, and number of LTCs, the estimated adjustment required is an increase of 13.5%.

For peer review only

Discussion

Summary

Using patient records from a sample of 150 Scottish general practices we demonstrate that substantially less care is received by patients in practices in socio-economically deprived areas compared to patients in affluent areas, but only when viewed in relation to morbidity, as measured by the number of LTCs. If general practices in highly SE deprived areas were to give an equivalent

For peer review only - http://bmjopen.bmj.com/site/about/guidelines.xhtml

amount of time (direct contact) to patients with the same level of need (the number of long-term conditions), they would require additional resources sufficient to support an increase of approximately 14% in patient contact time.

Strengths and limitations

This study utilises routine administrative data from a large population dataset which limits the potential bias associated with self-reported utilisation data[2] when seeking to measure unmet need. It demonstrates that data are retrievable from practice data systems to measure contact time with practice staff and allow for an estimation of resources required. The findings begin to address a universal problem about unmet need and resource allocation in primary care. However, our analysis excludes patients who made no appointments with their practice over the 3-year period. This may slightly bias our estimates of the average time spent with practice staff. However, such patients are likely to have very low health care need, and will have little impact on the resource requirements of their practices.

The accuracy of estimated time spent with patients has also not been ascertained in research, but we have reduced any inaccuracies by using record opening times to best reflect clinical contacts.[12] Outside the Quality and Outcomes Framework,[16] there is also some variation in how long-term health conditions are coded. However, we followed established methods to mitigate for potential errors.[6] We acknowledge that each long term condition included does not have equivalence in terms of impact on patients and that even within each condition there will be variation in the health care needs of individuals.[17]

The measure of socio-economic deprivation used is area-based, rather than one measured at an individual level to maximise accuracy; however this was the best available. Likewise we were unable – due to the anonymised nature of practice recruitment – to recruit a sample that was a perfect

For peer review only - http://bmjopen.bmj.com/site/about/guidelines.xhtml

representation of the population; however our TTP recruited practices based on our specific request to ensure an urban/rural and affluent/SE deprived area spread.

The lack of accurate information about ethnicity is a limitation. In our previous work,[11] we found that only 2.7% of appointments could be linked to ethnicity data in the medical records.

Given our aim of highlighting a potential source of unmet need, we used a simple division of practices into those serving relatively more SE deprived and affluent populations. Whether these findings hold true on a national scale, across the full spectrum of SE deprivation, remains to be seen.

Also, given the known association between deprivation and multimorbidity, our analysis focused on SE deprivation. Provision of medical care by general practices does have some distinct features depending on practice locality. For example, in remote rural settings practices provide more pre-hospital emergency care which means more time may be spent with patients in those circumstances and this may bias consultation time estimates in these areas. Future work will need to consider the impact of rurality on the associations we have observed.

Finally these data predate the rapid and far reaching changes to general practice access in the UK triggered by the Covid pandemic. It will be some time yet before the longer-term consequences and eventual access arrangements settle into a new normal and their consequences are understood.

Comparison with existing literature

Research from Scotland over 20 years ago, and more recently, observed that consultations in more socio-economically deprived areas tended to be shorter[18, 19] and these findings have been replicated in England.[20] We know that provision of GP resource is flatly distributed in Scotland[21] and there are indications that the revised Scottish GP contract funding formula from 2018 has not had a positive impact in more SE deprived areas.[22] An in-depth analysis conducted with English routine administrative data showed that practices in high SE deprivation areas have fewer GPs and more practice nurses than affluent area-based counterparts. This means GPs in those settings are caring for

For peer review only - http://bmjopen.bmj.com/site/about/guidelines.xhtml

10% more patients overall.[22] What this paper demonstrates, for the first time, is how clinically focussed evidence that relies on high quality data from a large percentage of the Scottish population can inform and improve models of care. Put simply, it appears that on average, a patient registered at a General Practice in Scotland serving an affluent population will receive more care from their practice than a patient of the same age, sex, and level of morbidity, registered with a practice serving a more SE deprived population.

Implications for research and/or practice

This study adds to growing evidence that provision of general practice care in the UK is not equitably distributed across practices and needs to change.[24] Patients with long-term health conditions living in the least affluent areas are losing out. This is unacceptable unmet need because it is systematically related to socio-economic factors.

Based on a relatively simple analysis using a sample of practices in Scotland, we estimate that a 14% increase in clinical staff contact time is required in SE deprived areas to equalise provision. A direct impact of this analysis is that governments, health service policy makers and planners should consider using data on contact time to measure the amount of care delivered to patients, as well as data on the number of recorded LTCs to estimate the level of need of GP practice populations. This is now achievable through routine administrative data capture and could be factored into resource allocation algorithms.

This paper adds to the large body of work now accumulated in the UK about the role general practice care has in perpetuating health inequalities and the inverse care law.[25] If our findings are replicated at a national level, using more complex analysis methods as used in deriving resource allocation formulas, then general practice could start to become part of the solution to socioeconomic health inequalities.

Conclusion

For peer review only

For peer review only - http://bmjopen.bmj.com/site/about/guidelines.xhtml

General practice provision in SE deprived areas needs to be better supported to deliver uniform care to patients with the same level of need across the UK. The analysis from this paper suggests that measuring LTCs and funding accordingly would make a significant contribution to achieving this aim.

Ethics approval

Letters of comfort were issued by the West of Scotland NHS Ethics Committee and the University of Glasgow College of Medical, Veterinary & Life Sciences Ethics Committee confirming that the full study did not need NHS ethics approval as this used routine data that had been extracted in an anonymised format. Public Benefit and Privacy Panel approval for use of the data was granted by NHS Information Services Scotland in December 2016.

Author contributions

AEW: Designed and managed the study, wrote first draft of manuscript, reviewed final version of manuscript. DE, PW: Designed and managed the study, reviewed final version of manuscript. RM: Processed data for analysis, reviewed final version of manuscript. AM: Conceived and carried out statistical analysis, wrote statistical methods and results, reviewed final version of manuscript.

Data Availability

These data were available from NHS Scotland and access permissions were contingent on them not being publicly available. New data access and TTP requests can be made to www.escro.co.uk for general practice data and https://www.isdscotland.org/products-and-services/edris/ to host the data securely and request data linkage. The authors had no special access permissions that others could not have. Analysis code is available from the authors upon reasonable request. The code will replicate the study findings, but the analysis can be replicated using other statistical software.

Acknowledgements

For peer review only

Thank you to all the GP practices who participated in the Serial Missed Appointments study and for strategic support from Ellen Lynch (Health and Social Care Analytical Services, Scottish Government). The general practice data expertise of Dave Kelly (Albasoft) was invaluable. Thanks also to the eDRIS team who facilitated the safe use of our data in the Safehaven, especially Dionysis Vragkos.

Study Protocol

See protocol paper.[12]

Funding

This work was supported by a grant from Chief Scientist Office, Scottish Government (reference CZH/4/1118) with Safe Haven and data linkage costs supported in lieu by the DSLS at Scottish Government. The funding sources for this analysis had no influence over study design, data collection, data analysis, data interpretation, the writing of the report, or the decision to submit for publication.

Competing Interests

None declared

Figure Legends

Figure 1: Mean duration of appointments (over 3 years), by age, sex, and number of long-term conditions, for practices with SE deprived and affluent populations.

For peer review only

For peer review only - http://bmjopen.bmj.com/site/about/guidelines.xhtml

References

1. Smith S, Connolly S. Re-thinking unmet need for health care: introducing a dynamic perspective. *Health Econ Policy Law*. 2020;15(4):440-57.
2. Allin S, Grignon M, Le Grand J. Subjective unmet need and utilization of health care services in Canada: What are the equity implications? *Soc Sci Med*. 2010;70(3):465-72.
3. Pearson-Stuttard J, Ezzati M, Gregg EW. Multimorbidity-a defining challenge for health systems. *Lancet Public Health*. 2019;4(12):e599-e600.
4. Whitty CJM, MacEwen C, Goddard A, et al. Rising to the challenge of multimorbidity. *BMJ*. 2020;368:l6964.
5. Rolewicz L, Keeble E, Paddison C, et al. Are the needs of people with multiple long-term conditions being met? Evidence from the 2018 General Practice Patient Survey. *BMJ Open*. 2020;10(11):e041569.
6. Barnett K, Mercer SMN, Watt G, et al. Epidemiology of Multi-morbidity and implications for health care, research and medical education: a cross sectional study. *Lancet*. 2012;380((9836)):37-43.
7. Menotti A, Mulder I, Nissinen A, et al. Prevalence of morbidity and multimorbidity in elderly male populations and their impact on 10-year all-cause mortality: The FINE study (Finland, Italy, Netherlands, Elderly). *J Clin Epidemiol*. 2001;54(7):680-6.
8. Stanley J, Semper K, Millar E, et al. Epidemiology of multimorbidity in New Zealand: a cross-sectional study using national-level hospital and pharmaceutical data. *BMJ Open*. 2018;8(5):e021689.
9. Sullivan MK, Jani BD, McConnachie A, et al. Hospitalisation events in people with chronic kidney disease as a component of multimorbidity: parallel cohort studies in research and routine care settings. *BMC Med.* 2021;19(1):278.
10. Albasoft, role and responsibilities as an NHS Trusted Third Party [Available from: http://www.escro.co.uk/marketing/TTP%20in%20primary%20care%20summary.pdf.
11. Ellis DA, McQueenie R, McConnachie A, et al. Demographic and practice factors predicting repeated non-attendance in primary care: a national retrospective cohort analysis. *Lancet Public Health*. 2017;2.
12. Williamson AE, Ellis DA, Wilson P, et al. Understanding repeated non-attendance in health services: a pilot analysis of administrative data and full study protocol for a national retrospective cohort. *BMJ Open*. 2017;7.
13. McQueenie R, Ellis DA, McConnachie A, et al. Morbidity, mortality and missed appointments in healthcare: a national retrospective data linkage study. *BMC Med*. 2019;17(1):2.
14. Scottish Index of Multiple Deprivation 2012 [Internet]. Scottish Government. 2012. Available from: http://www.gov.scot/Topics/Statistics/SIMD/DataAnalysis/Background-Data-2012.
15. R Core Team (2021). R: A language and environment for statistical computing. R Foundation for Statistical Computing, Vienna, Austria. URL https://www.R-project.org/.
16. Information Services Division NHS Scotland. General Practice Quality and Outcomes Framework UK: Public Health Scotland; 2020 [Available from: https://www.isdscotland.org/health-topics/general-practice/quality-and-outcomes-framework/.
17. Johnston MC, Crilly M, Black C, et al. Defining and measuring multimorbidity: a systematic review of systematic reviews. *Eur J Public Health*. 2019;29(1):182-9.
18. Stirling AM, Wilson P, McConnachie A. Deprivation, psychological distress, and consultation length in general practice. *Br J Gen Pract*. 2001;51(467):456-60.
19. Mercer SW, Watt GCM. The inverse care law: clinical primary care encounters in deprived and affluent areas of Scotland. *Ann Fam Med*. 2007;5(6):503-10.
20. Gopfert A, Deeny SR, Fisher R, et al. Primary care consultation length by deprivation and multimorbidity in England: an observational study using electronic patient records. *Br J Gen Pract*. 2021;71(704):e185-e92.

For peer review only

For peer review only - http://bmjopen.bmj.com/site/about/guidelines.xhtml

21. McLean G, Guthrie B, Mercer SW, et al. General practice funding underpins the persistence of the inverse care law: cross-sectional study in Scotland. *Br J Gen Pract*. 2015;65(641):e799-e805.

22. Morton C. Health Inequalities in Scotland A National Calamity; a Frontline GP view. UK: Independently published; 2020.

23. Fisher R, Dunn P, Asaria M, et al. Level or not? Comparing general practice in areas of high and low socioeconomic deprivation in England UK; 2020.

24. Ashworth M, L'Esperance V, Round T. Primary care funding entrenches health inequalities: time for a rethink. *Br J Gen Pract*. 2021;71(704):102-4.

25. Cookson R, Doran T, Asaria M, et al. The inverse care law re-examined: a global perspective. Lancet. 2021;397(10276):828-38.

For peer review only

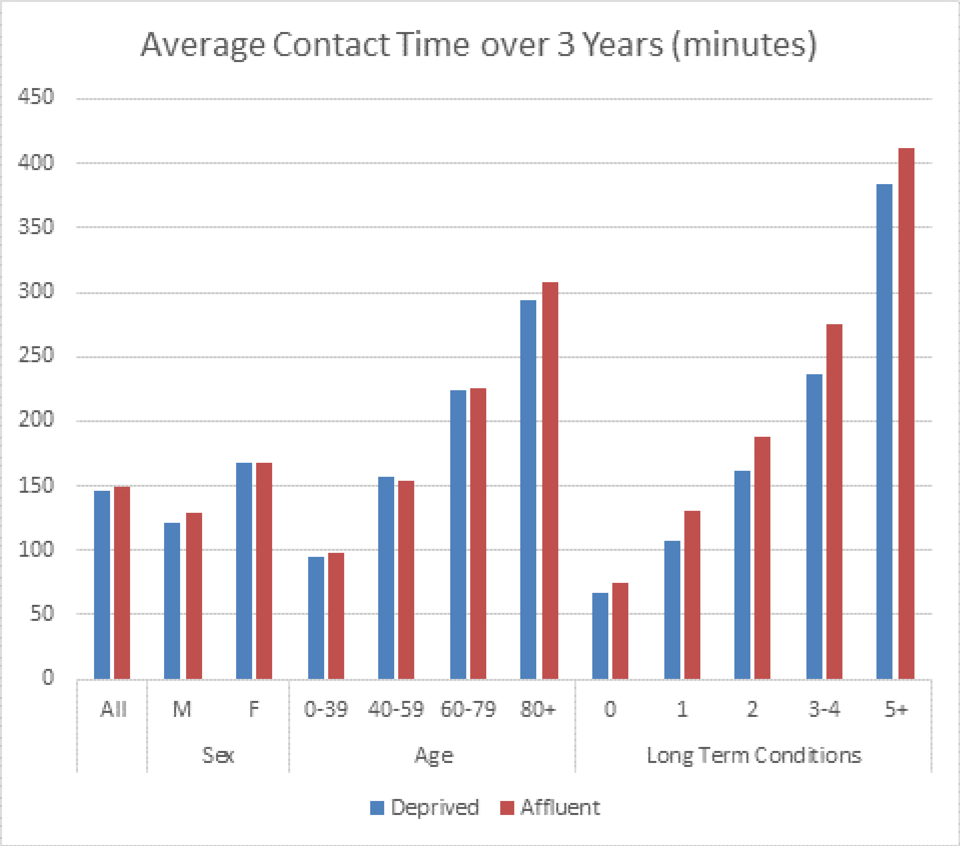

Mean duration of appointments (over 3 years), by age, sex, and number of long-term conditions, for practices with SE deprived and affluent populations.

127x111mm (96 x 96 DPI)

The RECORD statement – checklist of items, extended from the STROBE statement, that should be reported in observational studies using routinely collected health data.

	Item No.	STROBE items	Location in manuscript where items are reported	RECORD items	Location in manuscript where items are reported
**Title and abstract**					
	1	(a) Indicate the study's design with a commonly used term in the title or the abstract<br><br>(b) Provide in the abstract an informative and balanced summary of what was done and what was found	Title<br><br>p2, line 20-42	RECORD 1.1: The type of data used should be specified in the title or abstract. When possible, the name of the databases used should be included.<br><br>RECORD 1.2: If applicable, the geographic region and timeframe within which the study took place should be reported in the title or abstract.<br><br>RECORD 1.3: If linkage between databases was conducted for the study, this should be clearly stated in the title or abstract.	Title<br><br>p2, line 26-28<br><br>N/A
**Introduction**					
Background rationale	2	Explain the scientific background and rationale for the investigation being reported			p4, line 60-82
Objectives	3	State specific objectives, including any prespecified hypotheses			p4, line 80-82
**Methods**					
Study Design	4	Present key elements of study design early in the paper			Title<br>p5, line 84-96
Setting	5	Describe the setting, locations, and relevant dates, including periods of recruitment, exposure, follow-up, and data collection			p5, line 84-96

Participants	6	*(a) Cohort study* - Give the eligibility criteria, and the sources and methods of selection of participants. Describe methods of follow-up<br>*Case-control study* - Give the eligibility criteria, and the sources and methods of case ascertainment and control selection. Give the rationale for the choice of cases and controls<br>*Cross-sectional study* - Give the eligibility criteria, and the sources and methods of selection of participants	p5, line 84-103	RECORD 6.1: The methods of study population selection (such as codes or algorithms used to identify subjects) should be listed in detail. If this is not possible, an explanation should be provided.<br><br>RECORD 6.2: Any validation studies of the codes or algorithms used to select the population should be referenced. If validation was conducted for this study and not published elsewhere, detailed methods and results should be provided.	p5, line 91-95<br><br>N/A
		(b) Cohort study - For matched studies, give matching criteria and number of exposed and unexposed<br>*Case-control study* - For matched studies, give matching criteria and the number of controls per case	N/A	RECORD 6.3: If the study involved linkage of databases, consider use of a flow diagram or other graphical display to demonstrate the data linkage process, including the number of individuals with linked data at each stage.	N/A
Variables	7	Clearly define all outcomes, exposures, predictors, potential confounders, and effect modifiers. Give diagnostic criteria, if applicable.	p5-6, line 97-113	RECORD 7.1: A complete list of codes and algorithms used to classify exposures, outcomes, confounders, and effect modifiers should be provided. If these cannot be reported, an explanation should be provided.	p16, line 274-275
Data sources/ measurement	8	For each variable of interest, give sources of data and details of methods of assessment (measurement).<br>Describe comparability of assessment methods if there is more than one group			p5-6, line 84-113

For peer review only - http://bmjopen.bmj.com/site/about/guidelines.xhtml

Bias	9	Describe any efforts to address potential sources of bias			p13-14, line 197-224
Study size	10	Explain how the study size was arrived at			N/A
Quantitative variables	11	Explain how quantitative variables were handled in the analyses. If applicable, describe which groupings were chosen, and why			p5, line 98-103
Statistical methods	12	(a) Describe all statistical methods, including those used to control for confounding<br>(b) Describe any methods used to examine subgroups and interactions<br>(c) Explain how missing data were addressed<br>(d) *Cohort study* - If applicable, explain how loss to follow-up was addressed<br>*Case-control study* - If applicable, explain how matching of cases and controls was addressed<br>*Cross-sectional study* - If applicable, describe analytical methods taking account of sampling strategy<br>(e) Describe any sensitivity analyses			p6, line 114-119
Data access and cleaning methods		..		RECORD 12.1: Authors should describe the extent to which the investigators had access to the database population used to create the study population.	p5, line 97

For peer review only

				RECORD 12.2: Authors should provide information on the data cleaning methods used in the study.	p5, line 97
Linkage		..		RECORD 12.3: State whether the study included person-level, institutional-level, or other data linkage across two or more databases. The methods of linkage and methods of linkage quality evaluation should be provided.	N/A
**Results**					
Participants	13	(a) Report the numbers of individuals at each stage of the study (*e.g.*, numbers potentially eligible, examined for eligibility, confirmed eligible, included in the study, completing follow-up, and analysed)<br>(b) Give reasons for non-participation at each stage.<br>(c) Consider use of a flow diagram	p6-7, line 128-133	RECORD 13.1: Describe in detail the selection of the persons included in the study (*i.e.,* study population selection) including filtering based on data quality, data availability and linkage. The selection of included persons can be described in the text and/or by means of the study flow diagram.	p6-7, line 128-133
Descriptive data	14	(a) Give characteristics of study participants (*e.g.*, demographic, clinical, social) and information on exposures and potential confounders<br>(b) Indicate the number of participants with missing data for each variable of interest<br>(c) *Cohort study* - summarise follow-up time (*e.g.*, average and total amount)			p7, line 133-139
Outcome data	15	*Cohort study* - Report numbers of outcome events or summary measures over time<br>*Case-control study* - Report numbers in each exposure			p10, line 146-168

For peer review only

		category, or summary measures of exposure<br>*Cross-sectional study* - Report numbers of outcome events or summary measures			
Main results	16	(a) Give unadjusted estimates and, if applicable, confounder-adjusted estimates and their precision (e.g., 95% confidence interval). Make clear which confounders were adjusted for and why they were included<br>(b) Report category boundaries when continuous variables were categorized<br>(c) If relevant, consider translating estimates of relative risk into absolute risk for a meaningful time period			p10-12, line 162-187
Other analyses	17	Report other analyses done—e.g., analyses of subgroups and interactions, and sensitivity analyses			p10-12, line 162-187
**Discussion**					
Key results	18	Summarise key results with reference to study objectives			p12-13, line 189-196
Limitations	19	Discuss limitations of the study, taking into account sources of potential bias or imprecision. Discuss both direction and magnitude of any potential bias	p13-14, line 197-227	RECORD 19.1: Discuss the implications of using data that were not created or collected to answer the specific research question(s). Include discussion of misclassification bias, unmeasured confounding, missing data, and changing eligibility over time, as they pertain to the study being reported.	p13-14, line 197-227
Interpretation	20	Give a cautious overall interpretation of results			p14, line 228-240

For peer review only

		considering objectives, limitations, multiplicity of analyses, results from similar studies, and other relevant evidence			
Generalisability	21	Discuss the generalisability (external validity) of the study results			p13-14, line 214-224
**Other Information**					
Funding	22	Give the source of funding and the role of the funders for the present study and, if applicable, for the original study on which the present article is based			p16-17, line 283-288
Accessibility of protocol, raw data, and programming code		..		RECORD 22.1: Authors should provide information on how to access any supplemental information such as the study protocol, raw data, or programming code.	p16, line 281-282<br>p16, line 269-275

*Reference: Benchimol EI, Smeeth L, Guttmann A, Harron K, Moher D, Petersen I, Sørensen HT, von Elm E, Langan SM, the RECORD Working Committee. The REporting of studies Conducted using Observational Routinely-collected health Data (RECORD) Statement. *PLoS Medicine* 2015; in press.

*Checklist is protected under Creative Commons Attribution (CC BY) license.

For peer review only
